# Insight into the Roles of Metal Loading on CO_2_ Photocatalytic Reduction Behaviors of TiO_2_

**DOI:** 10.3390/nano12030474

**Published:** 2022-01-29

**Authors:** Darika Permporn, Rattabal Khunphonoi, Jetsadakorn Wilamat, Pongtanawat Khemthong, Prae Chirawatkul, Teera Butburee, Weradesh Sangkhun, Kitirote Wantala, Nurak Grisdanurak, Jirapat Santatiwongchai, Pussana Hirunsit, Wantana Klysubun, Mark Daniel G. de Luna

**Affiliations:** 1 Department of Environmental Engineering, Khon Kaen University, Khon Kaen 40002, Thailand; p.darika@kkumail.com (D.P.); jetsadakorn.wilamat@gmail.com (J.W.); 2 Chemical Kinetics and Applied Catalysis Laboratory (CKCL), Faculty of Engineering, Khon Kaen University, Khon Kaen 40002, Thailand; Kitirote@kku.ac.th; 3 Research Center for Environmental and Hazardous Substance Management (EHSM), Faculty of Engineering, Khon Kaen University, Khon Kaen 40002, Thailand; 4 National Nanotechnology Center (NANOTEC), National Science and Technology Development Agency (NSTDA), Klong Luang, Pathum Thani 12120, Thailand; pongtanawat@nanotec.or.th (P.K.); s.weradesh@gmail.com (W.S.); jirapat.san@nanotec.or.th (J.S.); pussana@nanotec.or.th (P.H.); 5 Synchrotron Light Research Institute (Public Organization), Nakhon Ratchasima 30000, Thailand; prae@slri.or.th (P.C.); wantana@slri.or.th (W.K.); 6 Center of Excellence in Environmental Catalysis and Adsorption, Faculty of Engineering, Thammasat University, Pathum Thani 12120, Thailand; gnurak@engr.tu.ac.th; 7 Department of Chemical Engineering, University of the Philippines Diliman, Quezon City 1101, Philippines; mgdeluna@up.edu.ph

**Keywords:** CO_2_ photoreduction, artificial photosynthesis, photodeposition, TiO_2_, IMPS, charge recombination

## Abstract

The photocatalytic reduction of carbon dioxide (CO_2_) into value-added chemicals is considered to be a green and sustainable technology, and has recently gained considerable research interest. In this work, titanium dioxide (TiO_2_) supported Pt, Pd, Ni, and Cu catalysts were synthesized by photodeposition. The formation of various metal species on an anatase TiO_2_ surface, after ultraviolet (UV) light irradiation, was investigated insightfully by the X-ray absorption near edge structure (XANES) technique. CO_2_ reduction under UV-light irradiation at an ambient pressure was demonstrated. To gain an insight into the charge recombination rate during reduction, the catalysts were carefully investigated by the intensity modulated photocurrent spectroscopy (IMPS) and photoluminescence spectroscopy (PL). The catalytic behaviors of the catalysts were investigated by density functional theory using the self-consistent Hubbard *U*-correction (DFT+U) approach. In addition, Mott–Schottky measurement was employed to study the effect of energy band alignment of metal-semiconductor on CO_2_ photoreduction. Heterojunction formed at Pt-, Pd-, Ni-, and Cu-TiO_2_ interface has crucial roles on the charge recombination and the catalytic behaviors. Furthermore, it was found that Pt-TiO_2_ provides the highest methanol yield of 17.85 µmol/g_cat_/h, and CO as a minor product. According to the IMPS data, Pt-TiO_2_ has the best charge transfer ability, with the mean electron transit time of 4.513 µs. We believe that this extensive study on the junction between TiO_2_ could provide a profound understanding of catalytic behaviors, which will pave the way for rational designs of novel catalysts with improved photocatalytic performance for CO_2_ reduction.

## 1. Introduction

The severe adverse effects of global warming resulting from excessive carbon dioxide (CO_2_) emission arouses the need for urgent research into CO_2_ reduction. CO_2_ conversion to valued-added chemicals or fuels has gained enormous research interest as a game-changing technology for sustainable development [1,2,3,4,5]. Artificial photosynthesis, which mimics natural photosynthesis using renewable solar energy and water to convert CO_2_ to manageable chemicals while leaving oxygen as a by-product, has been considered as one of the most green and sustainable technologies [6,7,8]. This method is also particularly attractive due to its ability to convert CO_2_ to value-added hydrocarbons using ambient temperature and pressure [2,9]. Several semiconductors, including TiO_2_ [10,11,12], CuO [13], g-C_3_N_4_ [14], Bi_2_WO_6_ [15], ZnO [16], SrTiO_3_ [17], and CeO_2_ [18], have been applied for photocatalytic reduction of CO_2_. Among these semiconductors, anatase TiO_2_ appears to be one of the most utilized catalysts due to its high performance, nontoxicity, high stability, and low cost [19,20,21]. However, rapid charge recombination is one of the important drawbacks, limiting the performance of TiO_2_, and its large band gap also results in low CO_2_ reduction efficiency [22].

Various strategies, such as surface modification, forming heterojunction and band alignment, and doping with metals and non-metals, have been reported as effective strategies to overcome these limitations and promote photocatalytic CO_2_ reduction performance [3,23,24,25,26]. In particular, metal-TiO_2_ composites have been shown to promote overall photocatalytic activity by reducing the recombination rate of the photogenerated charges and increasing light harvesting efficiency [27,28,29,30,31]. Generally, after irradiating the photocatalyst by incident light, the photogenerated electrons can transfer from the conduction band (CB) of photocatalysts across potential barriers to the contacting metal [32]. Therefore, metal acts as an electron sink for retarding the charge recombination rate. This can align the energy band between the metal and the semiconductor by shifting the Fermi level of the semiconductor to the metal located below the CB states of the semiconductor, and generating the semiconductor-metal heterojunction; namely the Schottky barriers [33]. The Schottky barrier effectively traps electrons, reducing the flow of electrons back to the semiconductor [23]. For example, Su et al. [3] studied the effect of Pd-loaded TiO_2_ on CO_2_ photoreduction. They found that the presence of Pd could enhance the CO_2_-to-methane conversion by around two orders of magnitude compared to the bare TiO_2_. It is well known that metal loading on semiconductors can enhance the photocatalytic CO_2_ reduction performance [3,22,34,35,36,37], however the roles and underlying mechanisms of metals remain unclear. Some intrinsic challenges and critical factors, including surface molecular structures, charge transfer behaviors, and charge recombination rate during reduction, are also debatable. Moreover, insights into the interaction of adsorbed CO_2_ with the semiconductor-modified surface as the catalytic sites are still expected to be further explored as the structure and the cation sites on the modified Ti surface composition are also involved in the catalytic pathways and selectivity of products. They can lower the reaction barrier to activate CO_2_, and stabilize CO_2_ intermediates to enhance CO_2_ photoreduction.

In the present study, the roles of loading metals, including Pt, Pd, Ni, and Cu, prepared by photodeposition on TiO_2_ towards the photocatalytic reduction of CO_2_ are investigated in many aspects simultaneously (i.e., band alignment, plasmonic effects, charge recombination, charge transfer, and surface chemistry), in order to gain an insight into true catalytic behavior. The plasmonic metal-TiO_2_ nanostructures and their compositions are extensively characterized by various techniques, including X-ray absorption near edge structure (XANES), X-ray diffraction (XRD), transmission electron microscopy (TEM), UV-visible diffuse reflectance spectra (UV-Vis), and inductively coupled plasma–optical emission spectroscopy (ICP-OES). The influences of energy band alignment of different heterojunctions, charge recombination behaviors, and photonic efficiency of metal-TiO_2_ are insightfully studied by intensity-modulated photocurrent spectroscopy (IMPS) and photoluminescence spectroscopy (PL), compared to the pristine anatase TiO_2_. The interactions of CO_2_ on TiO_2_ and Pt-TiO_2_ photocatalysts were studied by CO_2_-TPD, combined with theoretical simulation by density function theory (DFT). Interestingly, Pt-TiO_2_ showed the best photocatalytic CO_2_-to-methanol performance among the metals studied (Pt, Pd, Ni, and Cu) with a methanol production rate of 17.85 µmol/g_cat_/h, which is among the top unassisted photocatalysts that have been reported for CO_2_-to-methanol conversion. The impressive performance is attributed to suppressed charge recombination, suitable band alignment, and appropriate surface chemistry.

## 2. Materials and Methods

### 2.1. Metal Deposited-Semiconductor Preparation

Metal-deposited TiO_2_ semiconductors were prepared by the photodeposition method at room temperature. Next, 0.2 g anatase TiO_2_ (98% TiO_2_, Loba Chemie PVT. Ltd., Mumbai, India) was suspended in 50 mL of aqueous 2-propanol solution (99.8% V.S. Chem House, Bangkok, Thailand) (50 vol%). The mixture was purged with N_2_. Various metal salts; namely, H_2_Cl_6_Pt.6H_2_O (37.50 wt%. Sigma-Aldrich, St. Louis, MO, USA), PdCl_2_ (99.999 wt% Sigma-Aldrich, Steinheim, Germany), Ni (NO_3_)_2_.6H_2_O (97 wt%. Sigma-Aldrich, Steinheim, Germany) and CuN_2_O_6._3H_2_O (99 wt%. Sigma-Aldrich, Steinheim, Germany) were used as a metal source for Pt, Pd, Ni, and Cu, respectively. 1 mL of 0.1 μM metal solutions was gradually added into the catalyst suspension. The photodeposition of metals on the semiconductor was carried out under UV illumination. The suspension was irradiated with a mercury lamp (125 W), with a main emission in the UV range at 365 nm and a light intensity of 3.42 mW/cm^2^ under continuous stirring for 2 h. The obtained samples were precipitated by centrifugation (4500 rpm for 10 min), and washed with DI water for two cycles. Then, the samples were dried at 103 °C for 24 h. The total metal content of each sample was determined by inductively coupled plasma–optical emission spectroscopy (ICP-OES) (Perkin Elmer, AVIO 200, Waltham, MA, USA) (listed in Table S1 of supplement).

### 2.2. Characterization of Photocatalysts

Transmission electron microscopy (TEM) (HF-3300, Hitachi, Japan) was used to observe the morphologies of the as-synthesized catalysts. UV-visible diffuse reflectance spectra (UV-DRs) (UV-3101PC, Shimadzu, Japan) was used to analyze the band gap energy of the samples. The oxidation states and species of the metals deposited on the surface of anatase TiO_2_ were investigated by X-ray absorption near edge structure (XANES). XANES measurements were carried out with the fluorescent mode at the beamline 1.1 W and beamline 8, Synchrotron Light Research Institute (SLRI), Nakhon Ratchasima, Thailand. The data reduction of XANES spectra was performed using ATHENA program. CO_2_ temperature programmed desorption (CO_2_-TPD, Chemisorption analyzer; ChemStar TPX, Quantachrome Instruments, Boynton Beach, FL, USA) was carried out to investigate the interaction of CO_2_ and the catalyst. Photoluminescence (PL) (Avaspec-2048TEC-USB2-2, Apeldoorn, The Netherlands) and Intensity-modulated photocurrent spectroscopy (IMPS) (Metrohm Autolab, Utrecht, The Netherlands) were used to determine the charge dynamics and recombination. IMPS was obtained using a Metrohm Autolab PGSTAT12. The modulation frequency ranged from 120 to 500 kHz. The Mott–Schottky technique was used to classify the semiconductor types, and also used to estimate the flat band potential *V_fb_* and band alignment of the composites. The Mott–Schottky was obtained using the frequency response analyzer (Metrohm Autolab PGSTAT204, Utrecht, The Netherlands) with an applied bias ranging from 1.5 to −1.0 V (vs Ag/AgCl), and the frequency of impedance was fixed at 1 kHz with the RMS amplitude of 10 mV.

The Mott–Schottky equation (Equation (1)) involves the relationship between the capacitance and the biased voltage across the semiconductor/electrolyte interface. The derived Mott–Schottky plots were fitted by using the simple linear regression method.

(1) $$\frac{1}{C_{SC}^{2}}=\frac{2}{\varepsilon_{0}\varepsilon_{r}eA^{2}N_{D}}\left(V-V_{fb}-\frac{k_{B}T}{e}\right)$$

where $C_{SC}^{2}$ is the space charge capacitance density (F). V is the applied potential (V). $\varepsilon_{0}$ (F·m^−1^) and $\varepsilon_{r}$ are the vacuum permittivity and relative permittivity of TiO_2_, respectively [38]. e and $k_{B}$ are electron charge (C) and Boltzmann’s constant (m^2^·kg·s^−2^·K^−1^), respectively. A and T are the active surface area (cm^2^) and the absolute temperature (K), respectively. $N_{D}$ is the electron carrier density or donor concentration (cm^−1^). Corresponding to this equation, the $V_{fb}$ can be extracted from the intercept between the extrapolated linear line and *x*-axis. In addition, $N_{D}$ was also evaluated from the slope of the equation [39]. The position of the Fermi level (*E_F_*) relative to the conduction energy (*E_C_*) can be calculated by using Equations (2) and (3):

(2) $$E_{C}-E_{F}=\frac{kT}{e}ln\left(\frac{N_{C}}{N_{D}}\right)$$

(3) $$N_{C}=2{\left(\frac{2\pi m_{e}^{*}kT}{h^{2}}\right)}^{3/2}$$

where $N_{c}$ is the effective density of state in the conduction band (cm^−3^). h is Plank’s constant (m^2^·kg·s^−1^). The $N_{c}$ was calculated by setting $m_{e}^{*}$ as $10m_{0}$ [40]. Where the $m_{e}^{*}$ and $m_{0}$ are the density of state effective mass for the electrons of anatase TiO_2_ and the mass of the free electron (kg), respectively.

### 2.3. CO_2_ Photoreduction

The CO_2_ photoreduction was carried out in a closed system under UV- light (Hg-125 W). In a typical procedure, 0.1 g of the photocatalyst was dispersed in 50 mL of DI water. Prior to starting the reaction, N_2_ (Linde, UHP 99.999%) gas was first purged for 30 min to remove air, then CO_2_ was subsequently flowed into the system for 30 min to ensure that all oxygen and N_2_ were removed. The pressure in the reactor was kept at 1 atm, and the UV-light was irradiated to start the reaction. The resulting products from the photocatalytic CO_2_ reduction were measured by a gas chromatograph (GC-14 Shimadzu, Kyoto, Japan) equipped with a flame ionization detector (FID, Porapak Q mesh 50/80 Column) and a thermal conductivity detector (TCD, GC-SCI 310C) to identify and quantify the products. The product selectivity is calculated as Equation (4):

(4) $$\%Selectivity=\frac{Xi\times 100}{\sum Xi}$$

where *Xi* is product yield, including CH_3_OH and CO.

### 2.4. Density Functional Theory (DFT) Calculations

The reduced TiO_2_ surface was modeled by creating an oxygen vacancy on the surface. The simulations were carried out by an efficient density functional theory using the self-consistent Hubbard *U*-correction (DFT+U) approach [41,42] implemented in the Vienna Ab initio Simulation Package (VASP) [43,44,45,46]. The DFT+U methodology has been known as an ad hoc method that improves the description of *d*-states of the transition metals (3*d*-orbital in case of Ti) by implementing *U*-correction, solving the underestimated electronic interactions problems and providing a more accurate estimation than the standard DFT method [47,48]. The applied U value of 3.5 eV for Ti atoms was selected based on other works, which have performed the calculations of CO_2_ adsorbed on reduced TiO_2_ surface with different values of *U* and showed comparable calculated results to the experiments [47,49]. The effective Projector Augmented Wave (PAW) pseudopotentials [50] were constructed to describe the electron exchange and correlation effects. The calculations were performed within the generalized gradient approximation (GGA) and the Perdew–Burke–Ernzerhof (PBE) functional [51]. The self-consistent (SCF) field tolerance and the ionic force convergence threshold were 1.0 × 10^−5^ eV and −0.01 eV/Å, respectively. The kinetic energy cut-off for the plane wave basis set was set to 500 eV. The Monkhorst–Pack mesh sampling [52] k-points of 2 × 2 × 1 was used. The Methfessel–Paxton scheme of order two with a value of the smearing parameter σ of 0.03 eV was employed and the spin-polarized calculations were carried out. Bader charge analysis was performed using VASP—VTST [53,54,55]. For geometry optimization, the coordinates of the atoms in the two bottom layers were kept fixed while the rest of the atoms were allowed to relax. A vacuum space between slabs of 15 Å was set. To construct a reduced surface, an O atom at the bridge site (2c-O) was removed [47,56,57]. The optimized metal clusters of tetramers Pt, Pd, Ni and Cu were located on the optimized reduced TiO_2_ surface, following the configurations proposed by the literature [46,58,59,60,61]. The 3 × 1 supercell of the anatase TiO_2_ (101) was constructed with six layers of the (101) surface (Ti_36_O_72_). To study the CO_2_ adsorption on M_4_-TiO_2_ surfaces, both bent and linear CO_2_ molecules were considered.

## 3. Results and Discussion

### 3.1. Characterization of Photocatalysts

**Figure 1:**
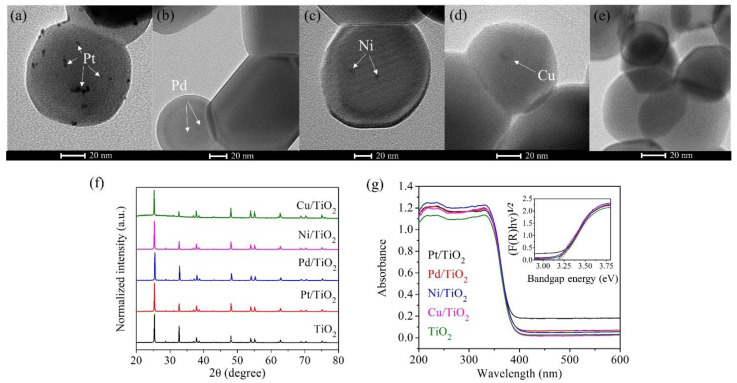
TEM images for (**a**) Pt-TiO_2_, (**b**) Pd-TiO_2_, (**c**) Ni-TiO_2_ (**d**) Cu-TiO_2_, (**e**) TiO_2_, (**f**) X-ray diffraction (XRD) (**g**) UV-vis spectra and bandgap energy (inset) of the photocatalysts with 0.1 wt% metal loading.

The particle sizes and morphologies of Pt, Pd, Ni, and Cu-loaded TiO_2_ prepared by photodeposition method and measured by Transmission Electron Microscopy (TEM) are shown in Figure 1a–e. As seen in the TEM images, both TiO_2_ and metal nanoparticles are in a spherical shape. The metal nanoparticles are in good distribution (Supplement Information, Figure S1). The average particle size of Pt, Pd, and Ni was approximately 4–5 nm, while Cu-TiO_2_ sample showed a larger particle size of (~12 nm). Moreover, there are also metal signals distributing throughout the whole samples, suggesting that there could be metals in other forms such as ions, clusters, or single atoms existing in the samples. The XRD patterns of the samples with different types of metal loaded on anatase TiO_2_ are displayed in Figure 1f. We found that the characteristic peaks of TiO_2_ (anatase, PDF 71-1167) were clearly observed, while the characteristic peaks belonging to Pt, Pd, Ni, and Cu species were invisible, due to the low concentration of the metals. The optical properties of the as-synthesized photocatalysts characterized by UV-Vis spectrophotometer are shown in Figure 1g. Their band gap energies were calculated using Kubelka–Munk equation derived from UV-visible diffuse reflectance (UV-vis DRs) spectra. According to the diffuse reflectance spectra, it was found that all samples have quite similar light absorption edges of around 400 nm, which corresponds to the similar band gap of 3.2 eV based on the Kubelka–Munk equation (inset of Figure 1g).

**Figure 2:**
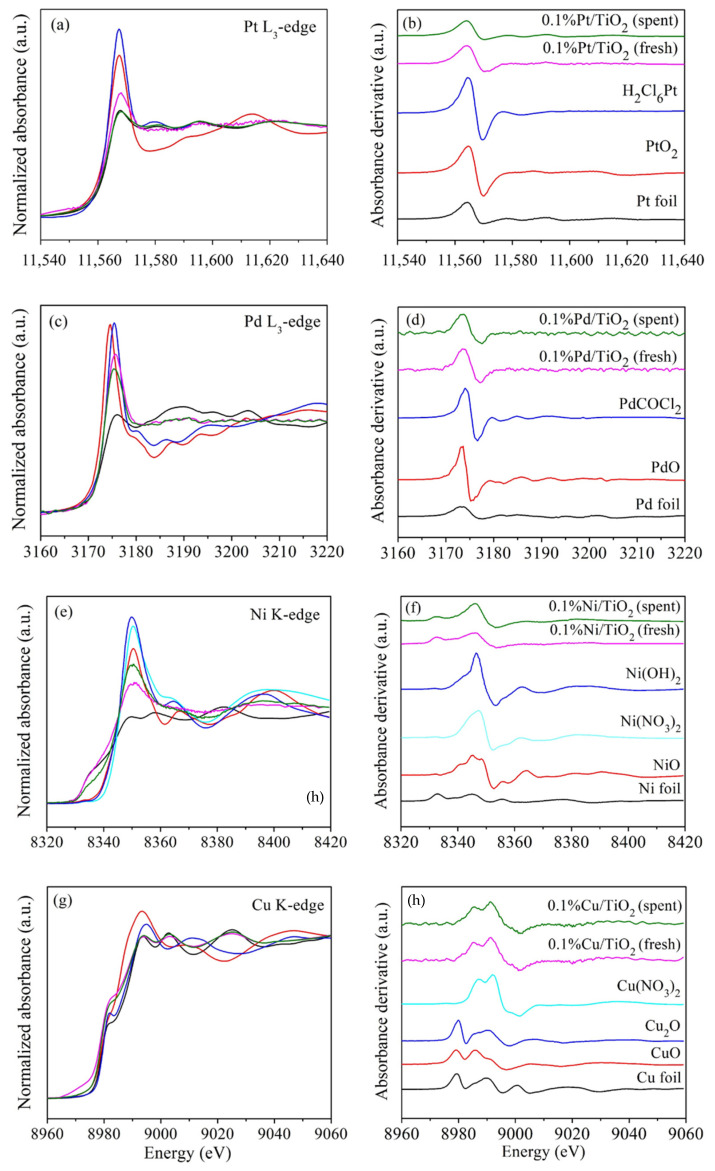
XANES spectra (**left**) and the first order derivative (**right**) of 0.1 wt% metals deposited on TiO_2_ photocatalysts Pt (**a**,**b**), Pd (**c**,**d**), Ni (**e**,**f**), and Cu (**g**,**h**) compared with reference standards.

The species and oxidation states of the fresh and spent metal-decorated TiO_2_ photocatalysts were further investigated by XANES. The XANES spectra accompanying the first order derivative of Pt-, Pd-, Ni-, and Cu-loaded TiO_2_ samples were compared to the standards illustrated in Figure 2. Figure 2a,b demonstrate the XANES spectra and the first order derivative of platinum samples (Pt L_3_-edge), compared to the spectra of H_2_Cl_6_Pt precursor and the reference standard materials, including Pt foil and PtO_2_ (representing the oxidation states of 0 and +4, respectively). Normally, Pt^0^ exhibits absorption edges at 11,567.9, while Pt^4+^ provides the edge energy at 11,567.4 eV (Table S2). We observed that the absorption edges of the fresh and spent Pt-TiO_2_ revealed the edge energy was close to that of the Pt^0^, indicating that both the fresh and spent Pt-TiO_2_ catalysts were mainly metallic [62]. As shown in Table 1, which tabulates the linear combination fit of the XANES spectra, the metallic form of Pt in the fresh sample was 72.4%, while that of the spent Pt-TiO_2_ was 100%. This evidence suggests that some of Pt^4+^ could be further reduced to form metallic Pt during the CO_2_ reduction.

Considering the Pd-decorated TiO_2_ samples, the Pd L_3_-edge XANES spectra and their first order derivative were compared with Pd foil, PdO, and PdCOCl_2_ (Figure 2c,d). The edge energies of the fresh and spent Pd-TiO_2_ were found to be 3175.4 and 3175.5 eV, which are significantly close to that of Pd foil, confirming the metallic Pd^0^ oxidation state [63]. This result is also consistent with the result from linear combination analysis, which shows 82.2% and 90.5% of Pd^0^ in the fresh and spent Pd-TiO_2_ samples, respectively. Figure 2e,f display the XANES spectra and the first order derivative of the Ni-loaded TiO_2_ samples, compared with the nickel standard references; namely, Ni foil, NiO, Ni(OH)_2_, and Ni(NO_3_)_2_ which have the edge energies of 8340.9, 8350.4, 8349.9, and 8350.4 eV, respectively [64]. The pre-edge of both fresh and spent Ni-TiO_2_ were found at 8341.2 eV and 8339.9 eV, respectively. The linear combination fitting of Ni-TiO_2_ samples shows that the Ni species in the fresh and the spent Ni-loaded TiO_2_ were close to Ni foil, confirming the existence of Ni^0^ in these samples.

Figure 2g shows the Cu K edge XANES spectra of the Cu-loaded TiO_2_ samples, which are compared to Cu foil, CuO, and Cu_2_O standards. Interestingly, the XANES features of both fresh and spent Cu-loaded TiO_2_ show similar edge energy positions, which are 8995.4 and 8995.8 eV, respectively. The linear combination analysis, as indicated in Figure 2g shows unclear species of Cu. The presence of mixed oxidation states of the CuO and Cu_2_O can be clarified by the first derivatives of the absorption edges, as shown in Figure 2h. We observed that Cu^1+^ and Cu^2+^ components existed in both samples while the metallic copper disappeared. According to the investigation of the metal oxidation states by XANES from the linear combination analysis as tabulated in Table 1, it can be noted that the metal nanoparticles resulting from photodeposition in the fresh and spent Pt-, Pd-, and Ni-loaded TiO_2_ were mostly in metallic form. However, Cu-TiO_2_ favorably formed Cu^1+^ species with a ratio of 60.1 and 93.2% for fresh and spent Cu-TiO_2_, respectively.

Previous research has explored the metallic behaviors on the photodeposition and photo-oxidation of propanol using the photodeposition methodology [65]. The proposed mechanism for metal photodeposited TiO_2_ is given in Equations (5)–(7). *H^+^* is a proton produced by the photo-oxidation of propanol with holes, as shown in Equation (6). Metal ions were reduced over TiO_2_ by reacting with photogenerated electrons, resulting in the formation of metallic particles, as validated by XANES:

(5) $$TiO_{2}+hv\to TiO_{2}(e^{-}{}_{CB}+h^{+}{}_{VB})$$

(6) C3H7OH+h+VB→C∗3H6OH+H+

(7) $$M^{n+}+ne^{-}{}_{CB}\to M(0)$$

**Figure 3:**
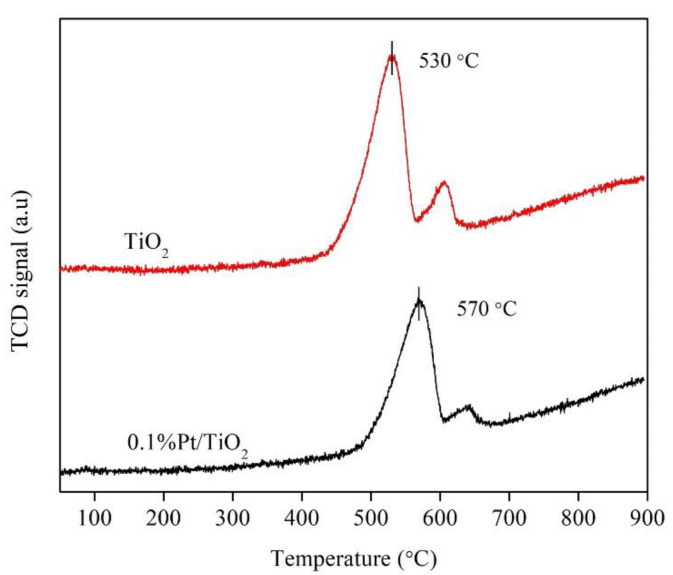
CO_2_-TPD profiles of TiO_2_ and Pt-deposited TiO_2_.

CO_2_ temperature programmed desorption (CO_2_-TPD) was carried out to investigate the interaction of CO_2_ reactant on the catalyst as shown in Figure 3 for the adsorption temperature from 50 to 900 °C. The main spectra can be assigned to the molecularly adsorbed bidentate carbonates ${(\mathrm{b-}\mathrm{HCO}}_{3}^{2-})$ (380–550 °C) and monodentate carbonate ${(\mathrm{m-}\mathrm{HCO}}_{3}^{2-})$ (550–760 °C), corresponding to strong basic sites of catalysts [66]. The peak intensity of ${\mathrm{b-}\mathrm{HCO}}_{3}^{2-}$ and ${\mathrm{m-}\mathrm{HCO}}_{3}^{2-}$ over Pt-TiO_2_ was lower than TiO_2_, suggesting that more medium and strong basic sites were formed over TiO_2_. However, as compared to pristine TiO_2_, the chemical desorption peaks of Pt-deposited TiO_2_ have shifted to a higher temperature, implying the stronger basicity of its adsorption sites [67]. The ${\mathrm{b-}\mathrm{HCO}}_{3}^{2-}$ and ${\mathrm{m-}\mathrm{HCO}}_{3}^{2-}$ species were generated from CO_2_ molecules combined with oxygen atoms or metal atoms of the cocatalyst [68].

### 3.2. CO_2_ Photocatalytic Reduction

**Figure 4:**
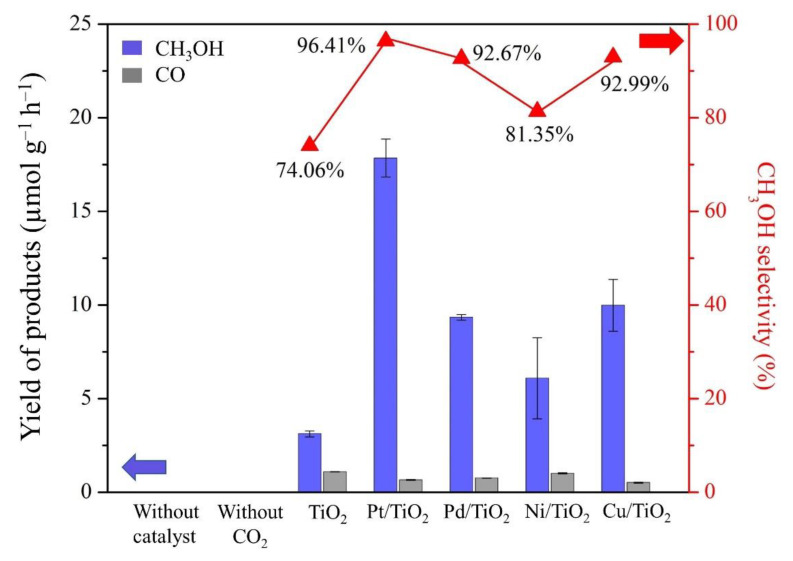
CH_3_OH and CO yields and selectivity from CO_2_ photocatalytic reaction over various M-TiO_2_ catalysts.

The photocatalytic reduction of CO_2_ was performed with liquid H_2_O under UV-light irradiation using various metal-loaded TiO_2_ samples as a photocatalyst. For the control experiment, the photocatalytic CO_2_ reduction was performed without photocatalysts and light irradiation. There was no reduced CO_2_ detected during the reaction, indicating that the reduced CO_2_ products were generated by photocatalytic reactions. The influence of the metals (Pt, Pd, Ni, and Cu) on CO_2_ photoreduction was carefully investigated, and the results are shown in Figure 4. The amount of metal deposited on TiO_2_-based photocatalyst was fixed at around 0.1 wt%, and this was confirmed by ICP-OES (Supplement Information, Table S1). Methanol and CO were the major and minor products, respectively (Figure S2). After 2 h photoreaction, methanol was produced approximately 3.12 µmol/g_cat_/h over the pristine TiO_2_. We found that metal loading can significantly promote the generation of the reduced CO_2_ products, compared to the pristine TiO_2_. Different dopants (Pt, Pd, Ni, and Cu) on TiO_2_ resulted in different degrees of enhancement on the photocatalytic CO_2_ reduction performance. The highest amount of methanol yield was found over the Pt-TiO_2_ catalyst with the production rate of 17.85 µmol/g_cat_/h with selectivity of 96.41%, following by Cu, Pd, and Ni-TiO_2_, which can produce methanol of 9.98, 9.35, and 6.09 µmol/g_cat_/h, respectively (Figure 4). The CO production over all catalysts seems negligible, as no higher than 2.5 µmol/g_cat_/h of CO was detected in any samples. The possible products in the liquid phase, such as formaldehyde, were also undetectable, as analyzed by a UV-Vis spectrophotometer. Moreover, 0.1 wt% Pt-TiO_2_ was carried out on the photoreaction without the involvement of CO_2_ to verify methanol and CO produced by CO_2_ photoreduction over metal loaded TiO_2_. The result showed that no product was detected (Supplement Information, Figure S3). Furthermore, CHN analysis of 0.1 wt% Pt-TiO_2_ catalyst showed that the amount of carbon on the catalyst was negligible (Table S3). This evidence indicated that methanol and CO were produced by the photoreduction of CO_2_. Pt-TiO_2_ also showed excellent stability, as the catalyst can maintain >90% of its original reactivity after running for three cycles (Supplement Information, Figure S4 and Table S8).

To attain a greater understanding of CO_2_ adsorption and reaction on TiO_2_ supported catalysts loading with Pt, Pd, Ni, and Cu, we further computationally evaluated the structural and electronic characteristics of CO_2_ adsorption on the supported tetramer metal clusters, including Pt, Pd, Ni, and Cu on the reduced surface of anatase TiO_2_ (101). The anatase TiO_2_ (101) surface consists of five-fold (5c-Ti) and six-fold (6c-Ti) coordinated Ti atoms and two-fold (2c-O) and three-fold (3c-O) coordinated O atoms at the surface (see Figure S6). These tetrameric metal clusters well represent both two- and three-dimensional deposited clusters. Three possible CO_2_ adsorption sites found in this study could be categorized as: (i) CO_2_ binding on the metal cluster; (ii) CO_2_ binding at the interface between the metal cluster and the TiO_2_; and (iii) linear CO_2_ adsorbed on the TiO_2_ surface (Supplement Information, Figures S5–S9 and Tables S4–S7).

**Figure 5:**
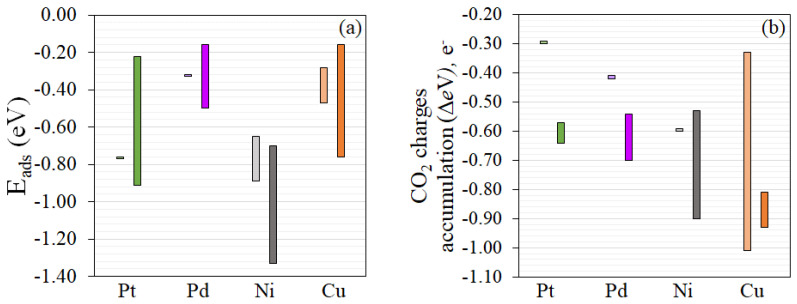
Calculated properties for CO_2_ adsorption on reduced anatase metal-TiO_2_ surfaces. (**a**) The adsorption energy ranges (**b**) CO_2_ charge ranges for different CO_2_ adsorption sites. The colors represent the different metals; green, purple, gray, and orange are Pt, Pd, Ni, and Cu, respectively. The light and dark shades of color represent CO_2_ adsorption sites at metal sites and at metal-TiO_2_ interface sites, respectively.

To analyze the CO_2_ adsorption systems, four properties of CO_2_ were analyzed, including the adsorption energy of CO_2_, the angle of O-C-O of adsorbed CO_2_, the charge accumulation on the adsorbed CO_2_ molecule, and the vibrational frequencies of the adsorbed CO_2_. The adsorption energy ($E_{ads}$) of CO_2_ was calculated according to Equation (8):

(8) $$E_{ads}=E_{Co_{2}/M_{4}-TiO_{2}}-E_{M_{4}-TiO_{2}}-E_{CO_{2\;\left(g\right)}}$$

where $E_{Co_{2}/M_{4}-TiO_{2}}$ is the total energy of CO_2_ adsorbed system, $E_{M_{4}-TiO_{2}}$ and $E_{CO_{2\;\left(g\right)}}$ are the total energy of the metal clusters located on the reduced *TiO*_2_ surfaces and the energy of isolated CO_2_, respectively. The results are illustrated in Figure 5. We found that the more negative $E_{ads}$ indicated the more stable CO_2_ adsorption configuration. The difference in Bader charge (∆*e*) of CO_2_ were the changes of CO_2_ atomic charges upon adsorption. The negative ∆*e* implied electron accumulation. The more negative ∆*e* indicated the adsorbed CO_2_ molecule gains more electrons. To confirm the key bond characteristics of adsorbed CO_2_ anion, the vibration frequency calculations were obtained. Three key vibrational modes of CO_2_ including symmetric (ν1), bending (ν2), and asymmetric (ν3) stretching modes were agreed to for other calculations for CO_2_ anion adsorption on *M*_4_-*TiO*_2_ surfaces [47,59,69].

These experimental results reveal that the reaction mechanism of CO_2_ photoreaction over various metal-deposited TiO_2_ photocatalysts could proceed through the carbene pathway. This research fits well with the work reported by Habisreutinger and co-workers [70]. CO_2_ photoreaction could be initiated through the chemisorbed CO_2_ molecules on the heterogeneous catalyst and form the adsorbed ${\mathrm{CO}}_{2}^{•-}$ species on the surface [71], confirmed by computational DFT result. This reaction is more likely to occur at the metal-TiO_2_ interfaces rather than on the pure TiO_2_ or the metal surfaces, as indicated by the more negative *E_ads_* values (Tables S4–S7). The simultaneous photogenerated holes react with adsorbed water or hydroxide ions ${\mathrm{OH}}_{ads}^{-}$ to generate oxygen and proton. Subsequently, the ${\mathrm{CO}}_{2}^{•-}$ reacts with the adsorbed $H^{•}$, which is produced by the reduction of $H^{+}$ before cleavage to form carbon monoxide and hydroxide ion (OH^−^) [67]. The adsorbed CO can desorb from the catalyst sites to produce CO as a product due to the weak CO adsorption of catalyst surface [72]. The adsorbed CO can also combine with an additional two electrons to form carbon residue on the surface, and then react with three $H^{•}$ radicals to form **˙**CH radical, carbene, and methyl radicals. Methyl radicals can further react with hydroxyl radical to form methanol [73]. As shown in Figure 5a, Pt and Cu provide moderately negative *E_ads,_* which could facilitate both the adsorption and desorption processes of CO_2_ molecules, while Ni provides very negative *E_ads_*. In principle, Cu should also show good CO_2_-to-methanol conversion performance, as it has suitable *E_ads_* and good charge accumulation on CO_2_ molecules. However, our experiment found that Cu can easily turn to copper oxide and, hence, Pt appears to be the most promising among the plasmonic metals studied in this work.

**Figure 6:**
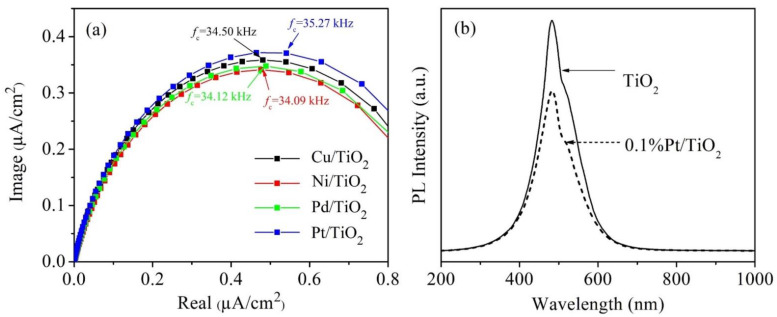
(**a**) IMPS of various metal-loaded TiO_2_ photocatalysts studied in this work, (**b**) Photoluminescence spectra (PL) of pristine TiO_2_ compared with Pt-TiO_2_ photocatalysts.

The enhancement of methanol yield from the modified TiO_2_ with metal loading could be explained by the formation of a Schottky barrier that could reduce *e*^−^/*h*^+^ recombination [3,34,74,75,76]. To gain insight into the charge recombination kinetics, intensity-modulated photocurrent spectroscopy (IMPS) and photoluminescence spectra (PL) were implemented to study the behaviors of charges. The IMPS results clarify the photogenerated charge transfer, as shown in Figure 6a. The frequency minimum in the complex plane of the IMPS plot can be used to calculate the mean transit time of the photogenerated *e*^−^ according to Equation (9) [77]:

(9) $$\tau_{c}=\frac{1}{2\pi fc}$$

where *f_c_* is the minimum point frequency (Hz) of the IMPS response. The smaller *τ_c_* indicates the better charge transfer [77]. The results showed that the *τ_c_* values of Pt-, Cu-, Pd-, and Ni-loaded TiO_2_ were 4.513, 4.613, 4.665, and 4.668 µs, respectively. Obviously, Pt-decorated TiO_2_ showed the lowest value of *τ_c_*, indicating enhanced charge transfer ability compared to other samples. In contrast, Ni-TiO_2_ has the highest values of *τ_c_*, indicating the poor charge transfer kinetics. These results are in good agreement with the actual photocatalytic CO_2_ reduction performance. Furthermore, PL was applied to further examine the charge transfer characteristics of Pt-TiO_2_ compared to the pristine TiO_2_, as shown in Figure 6b. The spectra of pure TiO_2_ showed higher PL intensity of the peak emission, indicating the higher charge recombination rate [78]. It can be noted that the presence of metals decorated on the TiO_2_ surface can enhance the photocatalytic reduction of CO_2_ by inhibiting the recombination rate [76].

**Figure 7:**
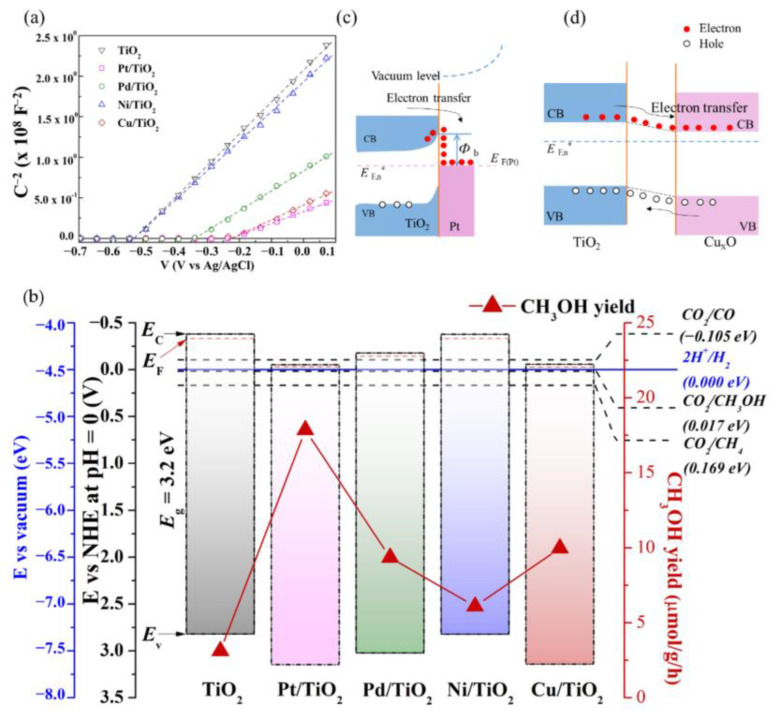
(**a**) Mott–Schottky plots of various photocatalyst films deposited onto FTO substrates measured at a frequency of 1 kHz in 0.1 M H_2_SO_4_ electrolyte solution (**b**) relationship between the CH_3_OH yield and the energy band alignment of TiO_2_ and Fermi levels of Pt, Pd, Ni and Cu based on Mott–Schottky results. (**c**) Schottky junction between Pt and TiO_2_. (**d**) the electron-hole pair separation in semiconductor-semiconductor heterojunction; $E_{F,n}^{*}$ is pseudo-Fermi level.

Figure 7a shows the Mott–Schottky plots of various synthesized photocatalysts deposited on FTO substrates. The linear regression of all metal-modified TiO_2_ samples showed a positive slope, indicating that the catalysts are n-type semiconductors. The *V_fb_* (vs Ag/AgCl) extracted from the Mott–Schottky results of TiO_2_, Pt-TiO_2_, Pd-TiO_2_, Ni-TiO_2_, and Cu-TiO_2_ are −0.53, −0.23, −0.34, −0.53, and −0.23 V, respectively. These *V_fb_* can be used to calculate the Fermi energy (*E_F_* vs. vacuum) of the catalysts. As shown in Table 2, Pt-TiO_2_ has the lowest *E_F_* (−4.47 eV), which is more negative than the unmodified TiO_2_ (−4.17 eV). Furthermore, it was found that the *E_F_* of the catalysts decreased as the work function (Φ_M_) of the deposited metals increased. It was believed that the higher work function of metal (low *E_F_*) can cause the more downward shifting of *E_F_* in TiO_2_.

The electron carrier density (*N_D_*) of Pt-TiO_2_ was calculated to be 3.27 × 10^20^ cm^−3^, which was higher than that of pristine TiO_2_. It is well known that a higher donor density implies a higher material conductivity, which facilitates the charge transport process [79]. Interestingly, Cu-TiO_2_ has low *E_F_* (−4.47 eV), which is comparable to Pt-TiO_2_ even though the work function (*Φ_M_*) of Cu is the lowest value (−4.65 eV) compared to the other interested metals. This might be due to the effect of Cu_2_O, which was the main component of Cu-TiO_2_ (see the linear combination analysis of XANES results). According to the report of Aguirre et al. [80], the *E_F_* of TiO_2_ can shift downward when it intimately contacted Cu_2_O to equilibrate the *E_F_* between two materials. Although the *E_F_* of Cu-TiO_2_ was equal to the *E_F_* of Pt-TiO_2_, the $N_{D}$ of Cu-TiO_2_ was lower (2.64 × 10^20^ cm^−3^). This could be the reason why Cu-TiO_2_ has lower CO_2_ reduction efficiency than that of Pt-TiO_2_.

Therefore, the relationship between the metal’s work function obtained from the Mott–Schottky results and the CH_3_OH yield is further analyzed as shown in Figure 7b. Normally, noble metals are known as efficient cocatalysts due to their large work functions. The metal’s work function (*Φ_M_*) is the energy needed to bring the electron from the metal’s Fermi energy to a vacuum level [33,81]. The larger metal work function, the better electron trapping ability. Band bending is formed when noble metals and semiconductors make intimate contact [32]. Pt-TiO_2_ showed the highest amount of methanol production, followed by Pd and Ni, respectively. This trend follows the order of the work function of the metals (see Figure 7b). The Schottky junction between Pt-decorated TiO_2_ with energy band alignment is shown in Figure 7c. The work function of Pt is −5.65 eV versus *E_vacuum_*, which is more positive than the conduction band of anatase TiO_2_ (−4.12 eV vs. vacuum) [82]. Therefore, electrons can easily transfer from the CB of TiO_2_ to Pt sites, which act as an electron sink [83]. On the other hand, metal with a smaller work function, such as Ni, causes a weaker driving force of electron migration [84]. As a result, the Pt-deposited on TiO_2_ sample showed significantly higher photocatalytic activity than the unloaded TiO_2_ due to the higher charge separation efficiency, which is also higher than the other metals [75,85]. However, the production of methanol was not in the trend over Cu-loaded TiO_2._ According to the XANES result, Cu loading on TiO_2_ was in a form of complex oxide. Therefore, the energy band alignment of Cu_x_O-TiO_2_ heterojunction was a semiconductor-semiconductor heterojunction, as revealed in Figure 7d. When Cu_x_O with lower CB level contacts with TiO_2_, which has higher level of CB, electrons in the CB of TiO_2_ can be transferred to that of Cu_x_O. The electrons and holes are transferred to the CB of Cu_x_O and the VB of TiO_2_, respectively [33]. As a result, the photoexcited electron-hole pairs can be separated by the electric field.

## 4. Conclusions

TiO_2_ loaded with various metals (Pt, Pd, Ni, and Cu) was successfully synthesized by photodeposition method. We found that all samples are active for CO_2_ photoreduction. On the other hand, various insightful characterizations reveal that Pt is the most promising metal, as it provides the largest work function when formed in heterojunction with TiO_2_, and provides the most appropriate CO_2_ adsorption and charge accumulation energies for methanol formation. which is also in good agreement with the experimental results. The large metal work function could enhance the charge transfer ability and suppress charge recombination. Electrons can transfer from the conduction band of a semiconductor to the metal surface and thus promote the photocatalytic reduction activity. When compared to Cu, Pd, and Ni-loaded TiO_2_ photocatalysts, Pt-loaded TiO_2_ photocatalyst also has the fastest charge transfer of 4.513 µs. Hence, Pt-TiO_2_ can generate methanol (major product) with the rate of 17.85 µmol/g_cat_/h, which is the highest photocatalytic CO_2_ reduction activity among the M-TiO_2_ that have been studied in this work, and among the top TiO_2_-based photocatalysts that have been reported for photocatalytic CO_2_-to-methanol conversion.

## Figures and Tables

**Table 1:** The linear combination analysis results of XANES spectra with standards.

Standards	Fresh (%)	Spent (%)
0.1%Pt/TiO_2_		
Pt foil	0.724	1.000
PtO_2_	0.121	0.000
H_2_Cl_6_Pt	0.155	0.000
0.1%Pd/TiO_2_		
Pd foil	0.822	0.905
PdO	0.178	0.095
0.1%Ni/TiO_2_		
Ni foil	0.970	0.828
NiO	0.000	0.000
Ni(OH)_2_	0.030	0.172
0.1%Cu/TiO_2_		
Cu foil	0.000	0.000
CuO	0.399	0.068
Cu_2_O	0.601	0.932

**Table 2:** The electronic properties derived from Mott–Schottky results.

Catalyst	*V_fb_* (V vs. Ag/AgCl)	*Φ_M_*	*E_F_*	*E_C_*	*E_V_*	*N_D_*	*E_CB_-E_F_*
		(eV)	(eV vs. Vacuum)			(cm^−3^)	(mV)
TiO_2_	−0.53	-	−4.17	−4.12	−7.32	1.23 × 10^20^	47.83
Pt/TiO_2_	−0.23	−5.65 (Pt)	−4.47	−4.45	−7.65	3.27 × 10^20^	22.60
Pd/TiO_2_	−0.34	−5.22 (Pd)	−4.36	−4.32	−7.52	1.96 × 10^20^	35.72
Ni/TiO_2_	−0.53	−5.04 (Ni)	−4.17	−4.12	−7.32	1.32 × 10^20^	45.98
Cu/TiO_2_	−0.23	−4.65 (Cu)	−4.47	−4.44	−7.64	2.64 × 10^20^	28.14

## Supplementary Materials

The following are available online at https://www.mdpi.com/article/10.3390/nano12030474/s1, Table S1: The actual amount of the metals in the samples at the theoretical content of 0.1% wt. measured by ICP-OES, Table S2: Edge energy of the samples and the standards obtained from XANES spectra, Table S3: Element of C, H, and N over Pt loaded TiO_2_, Table S4: Calculated properties of adsorption energy, O-C-O angle, CO_2_ charge accumulation^a^ and vibrational frequencies^b^ for CO_2_ adsorption on the V-Pt_4_-TiO_2_ structure, Table S5: Calculated properties of adsorption energy, O-C-O angle, CO_2_ charge accumulation^a^ and vibrational frequencies^b^ for CO_2_ adsorption on the V-Pd_4_-TiO_2_ structure, Table S6: Calculated properties of adsorption energy, O-C-O angle, CO_2_ charge accumulation^a^ and vibrational frequencies^b^ for CO_2_ adsorption on the V-Ni_4_-TiO_2_ structure, Table S7: Calculated properties of adsorption energy, O-C-O angle, CO_2_ charge accumulation^a^ and vibrational frequencies^b^ for CO_2_ adsorption on the V-Cu_4_-TiO_2_ structure, Table S8: Comparison of the photocatalysts for CO_2_-to-methanol conversion with this work [16,31,86,87,88,89,90], Figure S1: TEM-EDS result of photocatalysts, Figure S2: (a) GC-FID profile of the liquid products (b) GC-TCD profile of the gaseous products produced during the reaction, Figure S3: (a) GC-FID profile of the liquid products (b) GC-TCD profile of the gaseous products produced during the reaction without involvement of CO_2_ as reactant, Figure S4: CO_2_ photoreduction of the reuse catalyst of 0.1 wt% Pt-TiO_2_, Figure S5: The geometry of reduced anatase TiO_2_, and the most stable configurations of tetramer metal cluster adsorbed on reduced anatase TiO_2_. (Blue = Ti, Red = O, Green = Pt, Purple = Pd, Gray = Ni, and Orange = Cu), Figure S6: The geometry of CO_2_ adsorption configurations on the V-Pt_4_-TiO_2_ structure. (Blue = Ti, Red = O, Brown = C, Pink = O of CO_2_, and Green = Pt), Figure S7: The geometry of CO_2_ adsorption configurations on the V-Pd_4_-TiO_2_ structure. (Blue = Ti, Red = O, Brown = C, Pink = O of CO_2_, and Purple = Pd), Figure S8: The geometry of CO_2_ adsorption configurations on the V-Ni_4_-TiO_2_ structure. (Blue = Ti, Red = O, Brown = C, Pink = O of CO_2_, and Gray = Ni), Figure S9: The geometry of CO_2_ adsorption configurations on the V-Cu_4_-TiO_2_ structure. (Blue = Ti, Red = O, Brown = C, Pink = O of CO_2_, and Orange = Cu).

## References

[1] Duraccio V., Gnoni M.G., Elia V. (2015). Carbon capture and reuse in an industrial district: A technical and economic feasibility study. J. CO_2_ Util..

[2] Li D., Chen Y., Abanades S., Zhang Z. (2018). Enhanced activity of TiO_2_ by concentrating light for photoreduction of CO_2_ with H_2_O to CH_4_. Catal. Commun..

[3] Su K.Y., Chen C.Y., Wu R.J. (2019). Preparation of Pd/TiO_2_ nanowires for the photoreduction of CO_2_ into renewable hydrocarbon fuels. J. Taiwan Inst. Chem. Eng..

[4] Xie M., Qiu Y., Song C., Qi Y., Li Y., Kitamura Y. (2018). Optimization of Chlorella sorokiniana cultivation condition for simultaneous enhanced biomass and lipid production via CO_2_ fixation. Biores. Technol. Rep..

[5] Luo Y., Xia C., Abulizi R., Feng Q., Liu W., Zhang A. (2017). Electrocatalysis of CO_2_ reduction on nano silver cathode in ionic liquid BMIMBF4: Synthesis of dimethylcarbonate. Int. J. Electrochem. Sci..

[6] Gust D., Moore T.A., Moore A.L. (2009). Solar fuels via artificial photosynthesis. Acc. Chem. Res..

[7] Su J., Vayssieres L. (2016). A place in the sun for artificial photosynthesis?. ACS Energy Lett..

[8] Butburee T., Chakthranont P., Phawa C., Faungnawakij K. (2020). Beyond artificial photosynthesis: Prospects on photobiorefinery. ChemCatChem.

[9] Kumar A., Sharma G., Naushad M., Ahamad T., Veses R.C., Stadler F.J. (2019). Highly visible active Ag_2_CrO_4_/Ag/BiFeO_3_@RGO nano-junction for photoreduction of CO_2_ and photocatalytic removal of ciprofloxacin and bromate ions: The triggering effect of Ag and RGO. Chem. Eng. J..

[10] Butburee T., Kotchasarn P., Hirunsit P., Sun Z., Tang Q., Khemthong P., Sangkhun W., Thongsuwan W., Kumnorkaew P., Wang H. (2019). New understanding of crystal control and facet selectivity of titanium dioxide ruling photocatalytic performance. J. Mater. Chem. A.

[11] Butburee T., Sun Z., Centeno A., Xie F., Zhao Z., Wu D., Peerakiatkhajohn P., Thaweesak S., Wang H., Wang L. (2019). Improved CO_2_ photocatalytic reduction using a novel 3-component heterojunction. Nano Energy.

[12] You-Ji L., Wei C. (2011). Photocatalytic degradation of rhodamine B using nanocrystalline TiO_2_-zeolite surface composite catalysts: Effects of photocatalytic condition on degradation efficiency. Catal. Sci. Technol..

[13] Gusain R., Kumar P., Sharma O.P., Jain S.L., Khatri O.P. (2016). Reduced graphene oxide-CuO nanocomposites for photocatalytic conversion of CO_2_ into methanol under visible light irradiation. Appl. Catal. B.

[14] Sun Z., Wang S., Li Q., Lyu M., Butburee T., Luo B., Wang H., Fischer JM T.A., Zhang C., Wu Z. (2017). Enriching CO_2_ activation sites on graphitic carbon nitride with simultaneous introduction of electron-transfer promoters for superior photocatalytic CO_2_-to-fuel conversion. Adv. Sustain. Syst..

[15] Xiao L., Lin R., Wang J., Cui C., Wang J., Li Z. (2018). A novel hollow-hierarchical structured Bi_2_WO_6_ with enhanced photocatalytic activity for CO_2_ photoreduction. J. Colloid Interface Sci..

[16] Guo Q., Zhang Q., Wang H., Liu Z., Zhao Z. (2017). Unraveling the role of surface property in the photoreduction performance of CO_2_ and H_2_O catalyzed by the modified ZnO. Mol. Catal..

[17] Shao K., Wang Y., Iqbal M., Lin L., Wang K., Zhang X., He M., He T. (2018). Modification of Ag nanoparticles on the surface of SrTiO_3_ particles and resultant influence on photoreduction of CO_2_. Appl. Surf. Sci..

[18] Wang Y., Zhao J., Wang T., Li Y., Li X., Yin J., Wang C. (2016). CO_2_ photoreduction with H_2_O vapor on highly dispersed CeO_2_/TiO_2_ catalysts: Surface species and their reactivity. J. Catal..

[19] Sarkar A., Gracia-Espino E., Wågberg T., Shchukarev A., Mohl M., Rautio A.-R., Pitkänen O., Sharifi T., Kordas K., Mikkola J.P. (2016). Photocatalytic reduction of CO_2_ with H_2_O over modified TiO_2_ nanofibers: Understanding the reduction pathway. Nano Res..

[20] Phawa C., Prayoonpokarach S., Sinthiptharakoon K., Chakthranont P., Sangkhun W., Faungnawakij K., Butburee T. (2020). Effects of Matching Facet Pairs of TiO_2_ on Photoelectrochemical Water Splitting Behaviors. ChemCatChem.

[21] Xing Z., Zong X., Butburee T., Pan J., Bai Y., Wang L. (2016). Nanohybrid materials of titania nanosheets and plasmonic gold nanoparticles for effective hydrogen evolution. Appl. Catal. A.

[22] Xiong Z., Lei Z., Chen X., Gong B., Zhao Y., Zhang J., Zheng C., Wu J.C. (2017). CO_2_ photocatalytic reduction over Pt deposited TiO_2_ nanocrystals with coexposed {101} and {001} facets: Effect of deposition method and Pt precursors. Catal. Commun..

[23] Abdullah H., Khan M.M.R., Ong H.R., Yaakob Z. (2017). Modified TiO_2_ photocatalyst for CO_2_ photocatalytic reduction: An overview. J. CO_2_ Util..

[24] Schoonen M.A., Xu Y., Strongin D.R. (1998). An introduction to geocatalysis. J. Geochem. Explor..

[25] Chong R., Su C., Du Y., Fan Y., Ling Z., Chang Z., Li D. (2018). Insights into the role of MgAl layered double oxides interlayer in Pt/TiO_2_ toward photocatalytic CO_2_ reduction. J. Catal..

[26] Wang F., Jiang Y., Lawes D.J., Ball G.E., Zhou C., Liu Z., Amal R. (2015). Analysis of the promoted activity and molecular mechanism of hydrogen production over fine Au-Pt alloyed TiO_2_ photocatalysts. ACS Catal..

[27] Peerakiatkhajohn P., Butburee T., Yun J.-H., Chen H., Richards R.M., Wang L. (2015). A hybrid photoelectrode with plasmonic Au@TiO_2_ nanoparticles for enhanced photoelectrochemical water splitting. J. Mater. Chem. A.

[28] Butburee T., Bai Y., Pan J., Zong X., Sun C., Liu G., Wang L. (2014). Step-wise controlled growth of metal@TiO_2_ core–shells with plasmonic hot spots and their photocatalytic properties. J. Mater. Chem. A.

[29] Wang Y., Zu M., Li S., Butburee T., Wang L., Peng F., Zhang S. (2017). Dual modification of TiO_2_ nanorods for selective photoelectrochemical detection of organic compounds. Sens. Actuators B.

[30] Bai Y., Butburee T., Yu H., Li Z., Amal R., Lu G.M., Wang L. (2015). Controllable synthesis of concave cubic gold core–shell nanoparticles for plasmon-enhanced photon harvesting. J. Colloid Interface Sci..

[31] khalilzadeh A., Shariati A. (2019). Fe-N-TiO_2_/CPO-Cu-27 nanocomposite for superior CO_2_ photoreduction performance under visible light irradiation. Sol. Energy.

[32] Zhang Z., Yates J.T. (2012). Band bending in semiconductors: Chemical and physical consequences at surfaces and interfaces. Chem. Rev..

[33] Wang H., Zhang L., Chen Z., Hu J., Li S., Wang Z., Liu J., Wang X. (2014). Semiconductor heterojunction photocatalysts: Design, construction, and photocatalytic performances. Chem. Soc. Rev..

[34] Kočí K., Matějů K., Obalová L., Krejčíková S., Lacný Z., Plachá D., Čapek L., Hospodková A., Šolcová O. (2010). Effect of silver doping on the TiO_2_ for photocatalytic reduction of CO_2_. Appl. Catal. B.

[35] Li X., Zhuang Z., Li W., Pan H. (2012). Photocatalytic reduction of CO_2_ over noble metal-loaded and nitrogen-doped mesoporous TiO_2_. Appl. Catal. A.

[36] Liu D., Fernández Y., Ola O., Mackintosh S., Maroto-Valer M., Parlett C.M., Lee A.F., Wu J.C. (2012). On the impact of Cu dispersion on CO_2_ photoreduction over Cu/TiO_2_. Catal. Commun..

[37] Wei Y., Wu X., Zhao Y., Wang L., Zhao Z., Huang X., Liu J., Li J. (2018). Efficient photocatalysts of TiO_2_ nanocrystals-supported PtRu alloy nanoparticles for CO_2_ reduction with H_2_O: Synergistic effect of Pt-Ru. Appl. Catal. B.

[38] King D.M., Du X., Cavanagh A.S., Weimer A.W. (2008). Quantum confinement in amorphous TiO_2_ films studied via atomic layer deposition. Nanotechnology.

[39] Digdaya I.A., Han L., Buijs T.W.F., Zeman M., Dam B., Smets A.H.M., Smith W.A. (2015). Extracting large photovoltages from a-SiC photocathodes with an amorphous TiO_2_ front surface field layer for solar hydrogen evolution. Energy Environ. Sci..

[40] Enright B., Fitzmaurice D. (1996). Spectroscopic determination of electron and hole effective masses in a nanocrystalline semiconductor film. J. Phys. Chem..

[41] Anisimov V.I., Zaanen J., Andersen O.K. (1991). Band theory and Mott insulators: Hubbard *U* instead of stoner I. Phys. Rev. B.

[42] Dudarev S., Botton G. (1998). Electron-energy-loss spectra and the structural stability of nickel oxide: An LSDA+*U* study. Phys. Rev. B—Condens. Matter Mater. Phys..

[43] Kresse G., Hafner J. (1994). Ab initio molecular-dynamics simulation of the liquid-metalamorphous- semiconductor transition in germanium. Phys. Rev. B.

[44] Kresse G., Furthmüller J. (1996). Efficiency of ab-initio total energy calculations for metals and semiconductors using a plane-wave basis set. Comput. Mater. Sci..

[45] Kresse G., Furthmuller J. (1996). Efficient iterative schemes for ab initio total-energy calculations using a plane-wave basis set. Phys. Rev. B.

[46] Kresse G., Hafner J. (1993). Ab initio molecular dynamics for liquid metals. Phys. Rev. B.

[47] Yang C.T., Wood B.C., Bhethanabotla V.R., Joseph B. (2014). CO_2_ adsorption on anatase TiO_2_ (101) surfaces in the presence of subnanometer Ag/Pt clusters: Implications for CO_2_ photoreduction. J. Phys. Chem. C.

[48] Barcaro G., Thomas I.O., Fortunelli A. (2010). Validation of density-functional versus density-functional+U approaches for oxide ultrathin films. J. Chem. Phys..

[49] Sorescu D.C., Al-Saidi W.A., Jordan K.D. (2011). CO_2_ adsorption on TiO_2_ (101) anatase: A dispersion-corrected density functional theory study. J. Chem. Phys..

[50] Kresse G., Joubert D. (1999). From ultrasoft pseudopotentials to the projector augmented-wave method. Phys. Rev. B—Condens. Matter Mater. Phys..

[51] Perdew J.P., Burke K., Ernzerhof M. (1996). Generalized gradient approximation made simple. Phys. Rev. Lett..

[52] Monkhorst H.J., Pack J.D. (1976). Special points for Brillonin-zone integrations. Phys. Rev. B.

[53] Henkelman G., Arnaldsson A., Jónsson H. (2006). A fast and robust algorithm for bader decomposition of charge density. Comput. Mater. Sci..

[54] Sanville E., Kenny S.D., Smith R., Henkelman G. (2007). Improved grid-based algorithm for bader charge allocation. J. Comput. Chem..

[55] Tang W., Sanville E., Henkelman G. (2009). A grid-based bader analysis algorithm without lattice bias. J. Phys. Condens. Matter.

[56] Portillo-Vélez N.S., Olvera-Neria O., Hernández-Pérez I., Rubio-Ponce A. (2013). Localized electronic states induced by oxygen vacancies on anatase TiO_2_ (101) surface. Surf. Sci..

[57] He H., Zapol P., Curtiss L.A. (2010). A theoretical study of CO_2_ anions on anatase (101) surface. J. Phys. Chem. C.

[58] Yang C.T., Balakrishnan N., Bhethanabotla V.R., Joseph B. (2014). Interplay between subnanometer Ag and Pt clusters and anatase TiO_2_ (101) surface: Implications for catalysis and photocatalysis. J. Phys. Chem. C.

[59] Yang C.T., Wood B.C., Bhethanabotla V.R., Joseph B. (2015). The effect of the morphology of supported subnanometer Pt clusters on the first and key step of CO_2_ photoreduction. Phys. Chem. Chem. Phys..

[60] Meng L.D., Wang G.C. (2014). A DFT + U study of acetylene selective hydrogenation over anatase supported Pd_a_Ag_b_ (a + b = 4) cluster. Phys. Chem. Chem. Phys..

[61] Iyemperumal S.K., Deskins N.A. (2017). Activation of CO_2_ by supported Cu clusters. Phys. Chem. Chem. Phys..

[62] Bearden J.A., Burr A. (1967). Reevaluation of X-ray atomic energy levels. Rev. Mod. Phys..

[63] Dann E.K., Gibson E.K., Blackmore R.H., Catlow CR A., Collier P., Chutia A., Erden T.E., Hardacre C., Kroner A., Nachtegaal M. (2019). Structural selectivity of supported Pd nanoparticles for catalytic NH_3_ oxidation resolved using combined operando spectroscopy. Nat. Catal..

[64] Wirick S., Flynn G., Sutton S., Zolensky M. Comparison of nickel XANES spectra and elemental maps from a ureilite, a LL3. 8 ordinary chondrite, two carbonaceous chondrites and two large cluster IDPs. Proceedings of the 45th Lunar and Planetary Science Conference.

[65] Kunphonoi R., Afanasiev P., Geantet C., Puzenat E. Investigation on electron transfer from semiconductor to metal in photocatalytic H_2_ production. Proceedings of the 9th European meeting on Solar Chemistry and Photocatalysis: Environmental Applications (SPEA).

[66] Pu Y., Luo Y., Wei X., Sun J., Li L., Zou W., Dong L. (2019). Synergistic effects of Cu_2_O-decorated CeO_2_ on photocatalytic CO_2_ reduction: Surface Lewis acid/base and oxygen defect. Appl. Catal. B.

[67] Wang K., Zhang L., Su Y., Sun S., Wang Q., Wang H., Wang W. (2018). Boosted CO_2_ photoreduction to methane: Via Co doping in bismuth vanadate atomic layers. Catal. Sci. Technol..

[68] Wang Z., Zhou W., Wang X., Zhang X., Chen H., Hu H., Liu L., Ye J., Wang D. (2020). Enhanced photocatalytic CO_2_ reduction over TiO_2_ using metalloporphyrin as the cocatalyst. Catalysts.

[69] Cai S., Wang L., Heng S., Li H., Bai Y., Dang D., Wang Q., Zhang P., He C. (2020). Interaction of single-atom platinum-oxygen vacancy defects for the boosted photosplitting water H_2_ evolution and CO_2_ photoreduction: Experimental and theoretical study. J. Phys. Chem. C.

[70] Habisreutinger S.N., Schmidt-Mende L., Stolarczyk J.K. (2013). Photocatalytic reduction of CO_2_ on TiO_2_ and other semiconductors. Angew. Chem. Int. Ed..

[71] Usubharatana P., Mcmartin D., Veawab A., Tontiwachwuthikul P. (2006). Photocatalytic process for CO_2_ emission reduction from industrial flue gas streams. Ind. Eng. Chem. Res..

[72] Gattrell M., Gupta N., Co A. (2006). A review of the aqueous electrochemical reduction of CO_2_ to hydrocarbons at copper. J. Electroanal. Chem..

[73] Mori K., Yamashita H., Anpo M. (2012). Photocatalytic reduction of CO_2_ with H_2_O on various titanium oxide photocatalysts. RSC Adv..

[74] Gurunathan K. (2004). Photocatalytic hydrogen production using transition metal ions-doped γ-Bi_2_O_3_ semiconductor particles. Int. J. Hydrogen Energy.

[75] Jiang Z., Zhu J., Liu D., Wei W., Xie J., Chen M. (2014). In situ synthesis of bimetallic Ag/Pt loaded single-crystalline anatase TiO_2_ hollow nano-hemispheres and their improved photocatalytic properties. CrystEngComm.

[76] Umh H.N., Song C.K., Lee S.Y., Bae S., Kim T.Y., Kim Y.H., Joo J.B., Yi J. (2020). Band alignment modulations of metal-semiconductor system for enhanced charge separation directly related to a photocatalytic performance. Catal. Commun..

[77] Butburee T., Bai Y., Wang H., Chen H., Wang Z., Liu G., Zou J., Khemthong P., Lu GQ M., Wang L. (2018). 2D porous TiO_2_ single-crystalline nanostructure demonstrating high photo-electrochemical water splitting performance. Adv. Mater..

[78] Zhang Y., Yang X., Wang Y., Zhang P., Liu D., Li Y., Jin Z., Mamba B.B., Kuvarega A.T., Gui J. (2020). Insight into l-cysteine-assisted growth of Cu_2_S nanoparticles on exfoliated MoS_2_ nanosheets for effective photoreduction removal of Cr (VI). Appl. Surface Sci..

[79] Sariket D., Ray D., Baduri S., Ghosh S., Maity A., Bhattacharya C. (2020). Synthesis of g-C_3_N_4_/InVO_4_ Semiconductor for Improved Photocatalytic and Photoelectrochemical Applications. Electroanalysis.

[80] Aguirre M.E., Zhou R., Eugene A.J., Guzman M.I., Grela M.A. (2017). Cu_2_O/TiO_2_ heterostructures for CO_2_ reduction through a direct Z-scheme: Protecting Cu_2_O from photocorrosion. Appl. Catal. B Environ..

[81] Maicu M., Hidalgo M., Colón G., Navío J.A. (2011). Comparative study of the photodeposition of Pt, Au and Pd on pre-sulphated TiO_2_ for the photocatalytic decomposition of phenol. J. Photochem. Photobiol. A.

[82] Lu J., Jin H., Dai Y., Yang K., Huang B. (2011). Effect of electronegativity and charge balance on the visible-light-responsive photocatalytic activity of nonmetal doped anatase TiO_2_. Int. J. Photoenergy.

[83] Lee B.-Y., Park S.-H., Lee S.-C., Kang M., Park C.-H., Choung S.-J. (2003). Optical properties of Pt-TiO_2_ catalyst and photocatalytic activities for benzene decomposition. Korean J. Chem. Eng..

[84] Khan M.R., Chuan T.W., Yousuf A., Chowdhury M., Cheng C.K. (2015). Schottky barrier and surface plasmonic resonance phenomena towards the photocatalytic reaction: Study of their mechanisms to enhance photocatalytic activity. Catal. Sci. Technol..

[85] Xie S., Zhang Q., Liu G., Wang Y. (2016). Photocatalytic and photoelectrocatalytic reduction of CO_2_ using heterogeneous catalysts with controlled nanostructures. Chem. Commun..

[86] Tseng I.-H., Chang W.-C., Wu J.C.S. (2002). Photoreduction of CO_2_ using sol–gel derived titania and titania-supported copper catalysts. Appl. Catal. B Environ..

[87] Wu J.C., Lin H.-M., Lai C.-L. (2005). Photo reduction of CO_2_ to methanol using optical-fiber photoreactor. Appl. Catal. A Gen..

[88] Wang J.-J., Jing Y.-H., Ouyang T., Zhang Q., Chang C.-T. (2015). Photocatalytic reduction of CO_2_ to energy products using Cu–TiO_2_ /ZSM-5 and Co–TiO_2_/ZSM-5 under low energy irradiation. Catal. Commun..

[89] Zhang L., Li N., Jiu H., Qi G., Huang Y. (2015). ZnO-reduced graphene oxide nanocomposites as efficient photocatalysts for photocatalytic reduction of CO_2_. Ceram. Int..

[90] Song Y., Li J., Wang C. (2018). Modification of porphyrin/dipyridine metal complexes on the surface of TiO_2_ nanotubes with enhanced photocatalytic activity for photoreduction of CO_2_ into methanol. J. Mater. Res..

