# Supplementary material for: Insight into the Roles of Metal Loading on CO2 Photocatalytic Reduction Behaviors of TiO2
Source: Nanomaterials (Basel). 2022 Jan 29;12(3):474. doi: 10.3390/nano12030474 (PMC8839550; doi:10.3390/nano12030474)
Supplement: Supplementary file 1 [file nanomaterials-12-00474-s001.zip › nanomaterials-1553050-supplementary.pdf]

Supplement information

Table S1. The actual amount of the metals in the samples at the theoretical content of 0.1% wt. measured by ICP-OES

Samples	%metal from ICP-OES
Pt-TiO_2	0.10
Pd-TiO_2	0.07
Ni-TiO_2	0.10
Cu-TiO_2	0.09

Table S2. Edge energy of the samples and the standards obtained from XANES spectra

**samples**	**Edge energy (eV)**
Pt foil	11567.9
PtO_2	11567.4
H_2Cl_6Pt	11567.5
0.1%Pt/TiO_2 (fresh)	11568.1
0.1%Pt/TiO_2 (spent)	11568.2
Pd foil	3176.1
PdO	3174.6
$PdCOCl_2$	3175.4
0.1%Pd/TiO_2 (fresh)	3175.4
0.1%Pd/TiO_2 (spent)	3175.5
Ni foil	8340.9
NiO	8350.4
$Ni(OH)_2$	8349.9
$Ni(NO_3)_2$	8350.4
0.1%Ni/TiO_2 (fresh)	8341.2
0.1%Ni/TiO_2 (spent)	8339.9
Cu foil	8993.7
CuO	8983.4
Cu_2O	8982.0
0.1%Cu/TiO_2 (fresh)	8995.4
0.1%Cu/TiO_2 (spent)	8995.8

Table S3 Element of C, H, and N over Pt loaded TiO_2

Element	Composition (%)
C	0.44
H	0
N	0.29

Table S4 Calculated properties of adsorption energy, O─C─O angle, CO_2 charge accumulation [a]and vibrational frequencies[b] for CO_2 adsorption on the V-Pt_4-TiO_2 structure.

adsorption config.	E_{ads} (eV)	O─C─O (deg)	Δ*e* of CO_2			Sum *e* (CO_2)	ν(CO_2) (cm^{-1})		
			C	O	O		ν1	ν2	ν3
(i) CO_2 binding on the metal cluster									
V-Pt-1	-0.77	147.01	-0.41	0.00	0.11	-0.30	1139.79	636.44	1962.34
(ii) CO_2 binding at the interface between metal cluster and the TiO_2 surface									
V-Pt-2	-0.91	126.04	-0.53	-0.11	0.02	-0.61	1207.87	743.68	1522.55
V-Pt-3	-0.53	123.32	-0.72	-0.11	-0.02	-0.85	910.98	710.73	1602.41
V-Pt-4	-0.30	131.12	-0.58	-0.05	0.04	-0.59	1168.95	742.20	1718.81
V-Pt-5	-0.22	127.64	-0.54	-0.08	-0.02	-0.64	1099.63	759.01	1709.05
V-Pt-6	-0.22	128.83	-0.47	-0.08	-0.02	-0.57	1141.42	743.30	1725.75
(iii) linear CO_2 adsorbed on TiO_2 surface									
V-Pt-7	-0.22	178.45	0.02	-0.03	-0.01	-0.02	1340.58	602.35	2407.22

[a] Differences of Bader charge of CO_2 molecules between CO_2 adsorbed system and CO_2 molecule in gas-phase. Negative Δ*e* means electron accumulation after the adsorption.
[b] Vibrational frequencies of symmetric (ν1), bending (ν2), and asymmetric (ν3) stretching modes.

Table S5 Calculated properties of adsorption energy, O─C─O angle, CO_2 charge accumulation[a] and vibrational frequencies[b] for CO_2 adsorption on the V-Pd_4-TiO_2 structure.

adsorption config.	E_{ads} (eV)	O─C─O (deg)	Δ*e* of CO_2			Sum *e* (CO_2)	ν(CO_2) (cm^{-1})		
			C	O	O		ν1	ν2	ν3
(i) CO_2 binding on the metal cluster									
V-Pd-1	-0.33	143.43	-0.38	-0.07	0.02	-0.42	1193.34	641.13	1862.60
(ii) CO_2 binding at the interface between metal cluster and the TiO_2 surface									
V-Pd-2	-0.50	127.65	-0.63	-0.06	0.01	-0.67	1043.19	709.76	1608.24
V-Pd-3	-0.24	126.49	-0.66	-0.03	-0.01	-0.70	1105.02	719.00	1605.70
V-Pd-4	-0.16	134.65	-0.50	-0.09	0.05	-0.54	1167.47	708.98	1806.25
(iii) linear CO_2 adsorbed on TiO_2 surface									
V-Pd-5	-0.14	179.09	0.05	-0.04	0.00	0.01	1316.40	585.31	2365.22

[a] Differences of Bader charge of CO_2 molecules between CO_2 adsorbed system and CO_2 molecule in gas-phase. Negative Δ*e* means electron accumulation after the adsorption.
[b] Vibrational frequencies of symmetric (ν1), bending (ν2), and asymmetric (ν3) stretching modes.

Table S6 Calculated properties of adsorption energy, O─C─O angle, CO_2 charge accumulation [a] and vibrational frequencies[b] for CO_2 adsorption on the V-Ni_4-TiO_2 structure.

adsorption config.	E_{ads} (eV)	O─C─O (deg)	Δ*e* of CO_2			Sum *e* (CO_2)	ν(CO_2) (cm^{-1})		
			C	O	O		ν1	ν2	ν3
(i) CO_2 binding on the metal cluster									
V-Ni-1	-0.89	140.86	-0.58	-0.05	0.03	-0.60	1178.89	700.70	1779.26
V-Ni-2	-0.65	133.52	-0.58	0.01	-0.02	-0.60	1108.97	696.35	1724.51
(ii) CO_2 binding at the interface between metal cluster and the TiO_2 surface									
V-Ni-3	-1.33	125.64	-0.73	0.01	-0.06	-0.78	1187.25	739.31	1501.12
V-Ni-4	-0.80	143.06	-0.48	0.04	-0.09	-0.53	1190.11	690.47	1843.87
V-Ni-5	-0.70	130.34	-0.88	0.01	-0.03	-0.90	1063.47	691.32	1498.88
(iii) linear CO_2 adsorbed on TiO_2 surface									
V-Ni-6	-0.26	178.16	0.05	0.02	-0.07	-0.01	1327.14	591.97	2376.79

[a] Differences of Bader charge of CO_2 molecules between CO_2 adsorbed system and CO_2 molecule in gas-phase. Negative Δ*e* means electron accumulation after the adsorption.

[b] Vibrational frequencies of symmetric (ν1), bending (ν2), and asymmetric (ν3) stretching modes.

Table S7 Calculated properties of adsorption energy, O─C─O angle, CO_2 charge accumulation[a] and vibrational frequencies[b] for CO_2 adsorption on the V-Cu_4-TiO_2 structure.

adsorption config.	E_{ads} (eV)	O─C─O (deg)	Δ*e* of CO_2			Sum *e* (CO_2)	ν(CO_2) (cm^{-1})		
			C	O	O		ν1	ν2	ν3
(i) CO_2 binding on the metal cluster									
V-Cu-1	-0.47	135.33	-0.67	-0.01	-0.04	-0.71	1185.94	672.04	1731.43
V-Cu-2	-0.35	119.56	-0.88	-0.07	-0.06	-1.01	1102.06	751.79	1421.23
V-Cu-3	-0.28	149.86	-0.32	0.03	-0.05	-0.33	1224.57	557.65	2048.16
V-Cu-4	-0.76	123.80	-0.74	-0.06	-0.12	-0.93	1233.59	764.68	1471.91
(ii) CO_2 binding at the interface between metal cluster and the TiO_2 surface									
V-Cu-5	-0.19	130.33	-0.72	-0.07	-0.02	-0.81	1127.79	683.30	1585.79
V-Cu-6	-0.16	125.02	-0.71	-0.02	-0.08	-0.81	1046.77	737.53	1664.16
(iii) linear CO_2 adsorbed on TiO_2 surface									
V-Cu-7	-0.06	179.16	0.02	0.03	-0.07	-0.02	1353.14	620.75	2429.35

[a] Differences of Bader charge of CO_2 molecules between CO_2 adsorbed system and CO_2 molecule in gas-phase. Negative Δ*e* means electron accumulation after the adsorption.

[b] Vibrational frequencies of symmetric (ν1), bending (ν2), and asymmetric (ν3) stretching modes.

Table S8 Comparison of the photocatalysts for CO_2-to-methanol conversion with this work

Catalysts	Condition	Methanol yield (μmol $g^{-1}h^{-1}$)	Ref.
2.0% Cu/TiO_2	Hg lamp, 8W<br>CO_2 pressure= 1 bar<br>0.2 M NaOH as the electron donor	19.75	[1]
1.2 wt.% Cu/TiO_2	365 nm Hg lamp, 16W<br>pressure= 1.29 bar<br>H_2O as the electron donor	0.45	[2]
L-C/Z	Xe lamp, 450W<br>H_2O as the electron donor	6.78	[3]
Fe-N-Ti@50CPO	Hg lamp, 70 W<br>CO2 pressure= 0.758 bar<br>H_2O as the electron donor	2.17	[4]
Cu-TiO_2/ZSM-5	A 254 nm UV-lamp, 14 W<br>0.1 M $NaHCO_3$ as the electron donor	50.05	[5]
ZnO	Xenon lamp, 300W<br>1 M NaOH as the electron donor	17.45	[6]
P25	Xe lamp, 500W<br>0.05 M NaOH as the electron donor	33.22	[7]
**Pt-TiO_2**	**UV- light (Hg-125W)**<br>**CO_2 pressure= 1 atm**<br>**No electron donors/scarvengers**	**17.85**	**This work**

1.Tseng, I-H; Chang, W-C; Wu, J. C. S. Photoreduction of CO_2 using sol–gel derived titania and titania-supported copper catalysts. *Appl. Catal. B: Environ.* **2002**, *37*, 37–48.

2. Wu, J .C.; Lin, H-.M.; Lai, C-.L., Photo reduction of CO_2 to methanol using optical-fiber photoreactor .**2005,** *296*) 2(, 194-200.

3. Guo, Q.; Zhang, Q.; Wang, H.; Liu, Z.; Zhao, Z., Unraveling the role of surface property in the photoreduction performance of CO2 and H2O catalyzed by the modified ZnO *.Mol .Catal* .**2017,** *436*, 19-28.

4. Shariati, A., Fe-N-TiO_2/CPO-Cu-27 nanocomposite for superior CO_2 photoreduction performance under visible light irradiation *.Solar Energy* **2019,** *186*, 166-174.

5. Wang, J-.J.; Jing, Y-.H.; Ouyang, T.; Zhang, Q.; Chang, C-.T., Photocatalytic reduction of CO_2 to energy products using Cu–TiO_2/ZSM-5 and Co–TiO_2/ZSM-5 under low energy irradiation *.Catal .Commun* .**2015,** *59*, 69-72.

6. Zhang, L.; Li, N.; Jiu, H.; Qi, G.; Huang, Y., ZnO-reduced graphene oxide nanocomposites as efficient photocatalysts for photocatalytic reduction of CO_2 *.Ceram .Int* .**2015,** *41*) 5(, 6256-6262.

7. Song, Y.; Li, J.; Wang, C., Modification of porphyrin/dipyridine metal complexes on the surface of TiO_2 nanotubes with enhanced photocatalytic activity for photoreduction of CO_2 into methanol. *J. Mater. Res.* **2018,** *33* (17), 2612-2620.

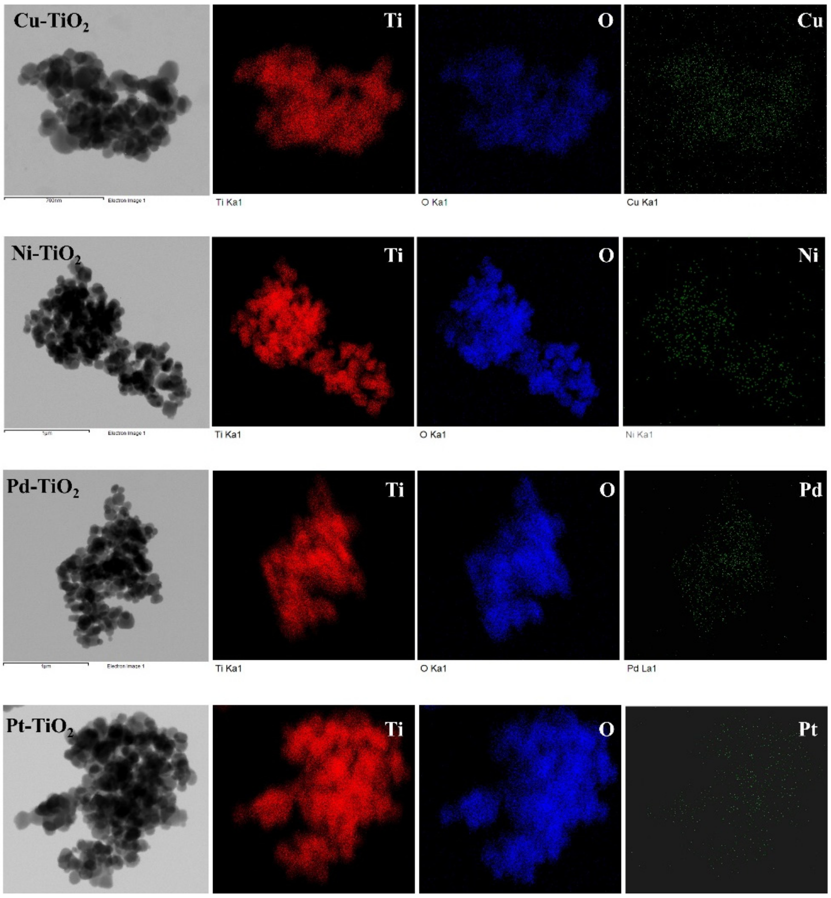

Figure S1 TEM-EDS result of photocatalysts.

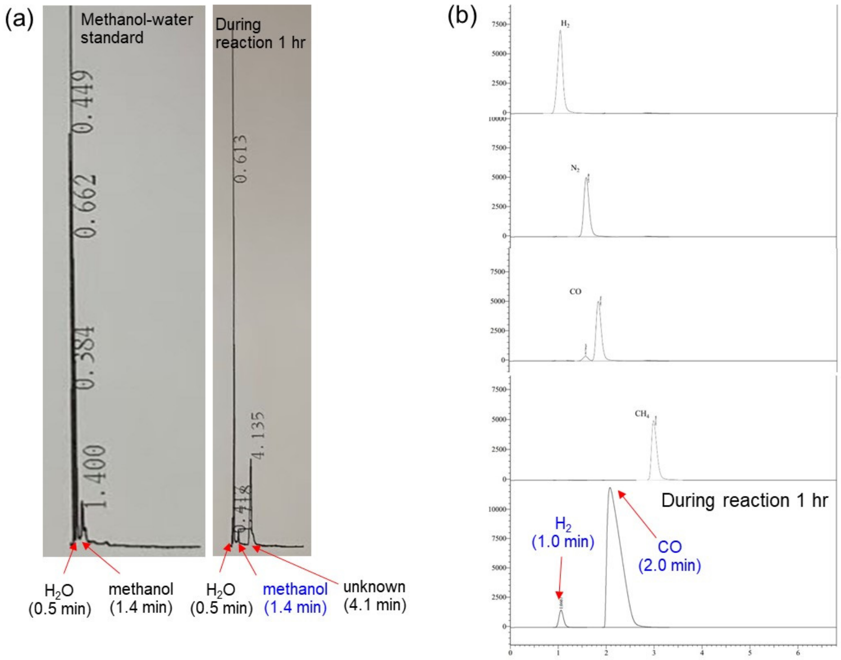

Figure S2 (a) GC-FID profile of the liquid products (b) GC-TCD profile of the gaseous products produced during the reaction.

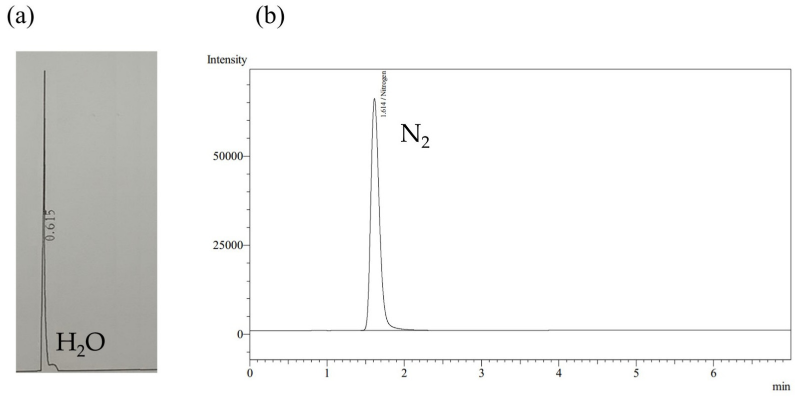

Figure S3 (a) GC-FID profile of the liquid products (b) GC-TCD profile of the gaseous products produced during the reaction without involvement of CO_2 as reactant.

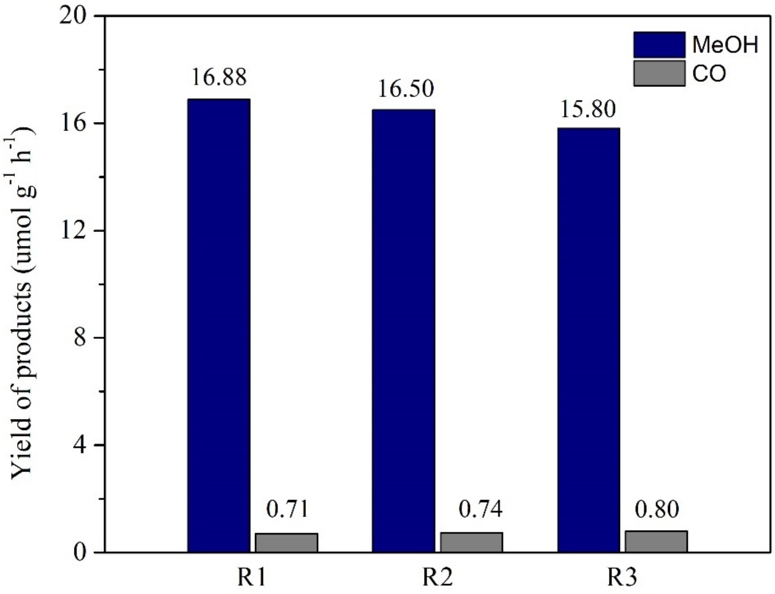

Figure S4 CO_2 photoreduction of the reuse catalyst of 0.1 wt% Pt-TiO_2.

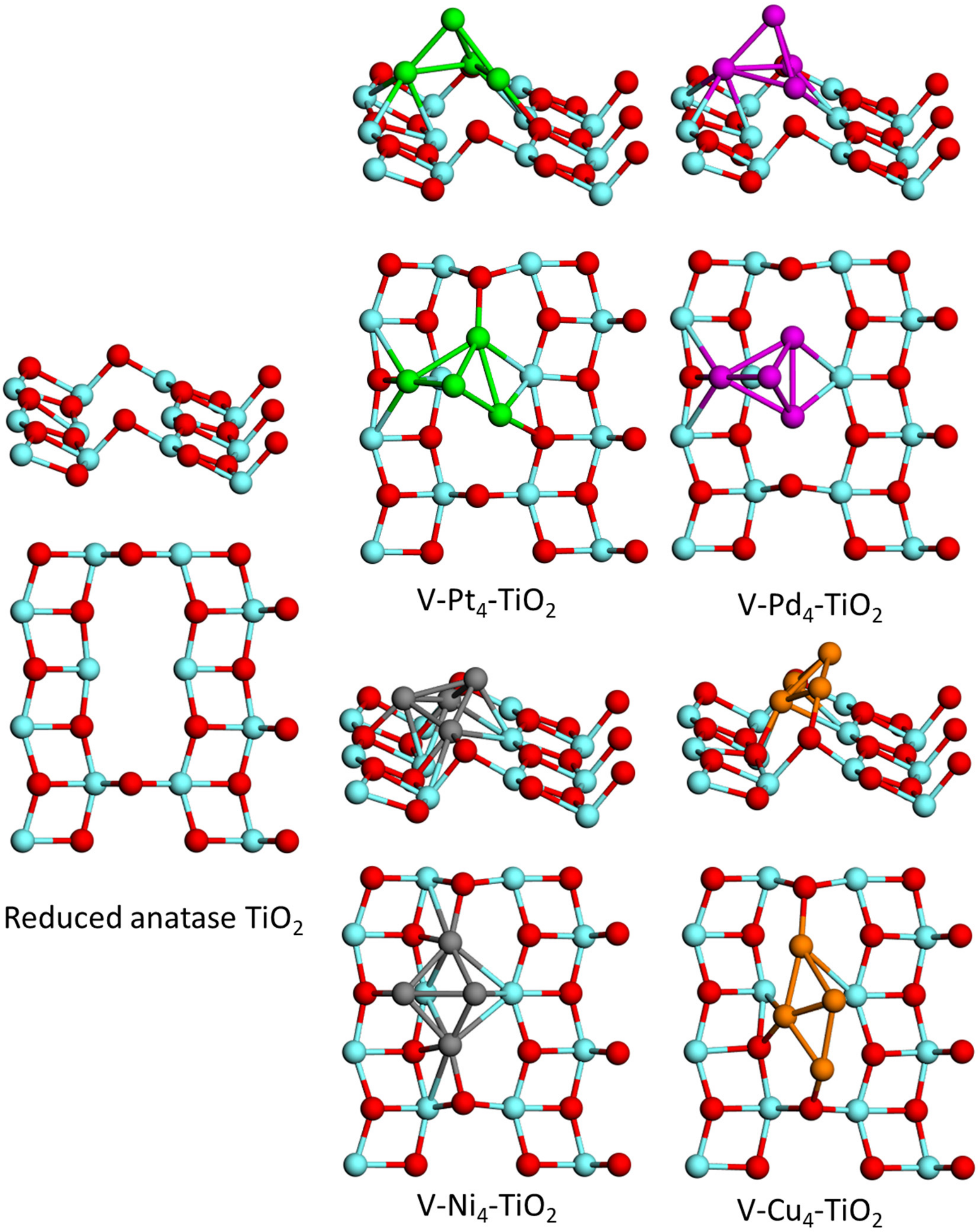

Figure S5 The geometry of reduced anatase TiO_2, and the most stable configurations of tetramer metal cluster adsorbed on reduced anatase TiO_2. (Blue = Ti, Red = O, Green = Pt, Purple = Pd, Gray = Ni, and Orange = Cu)

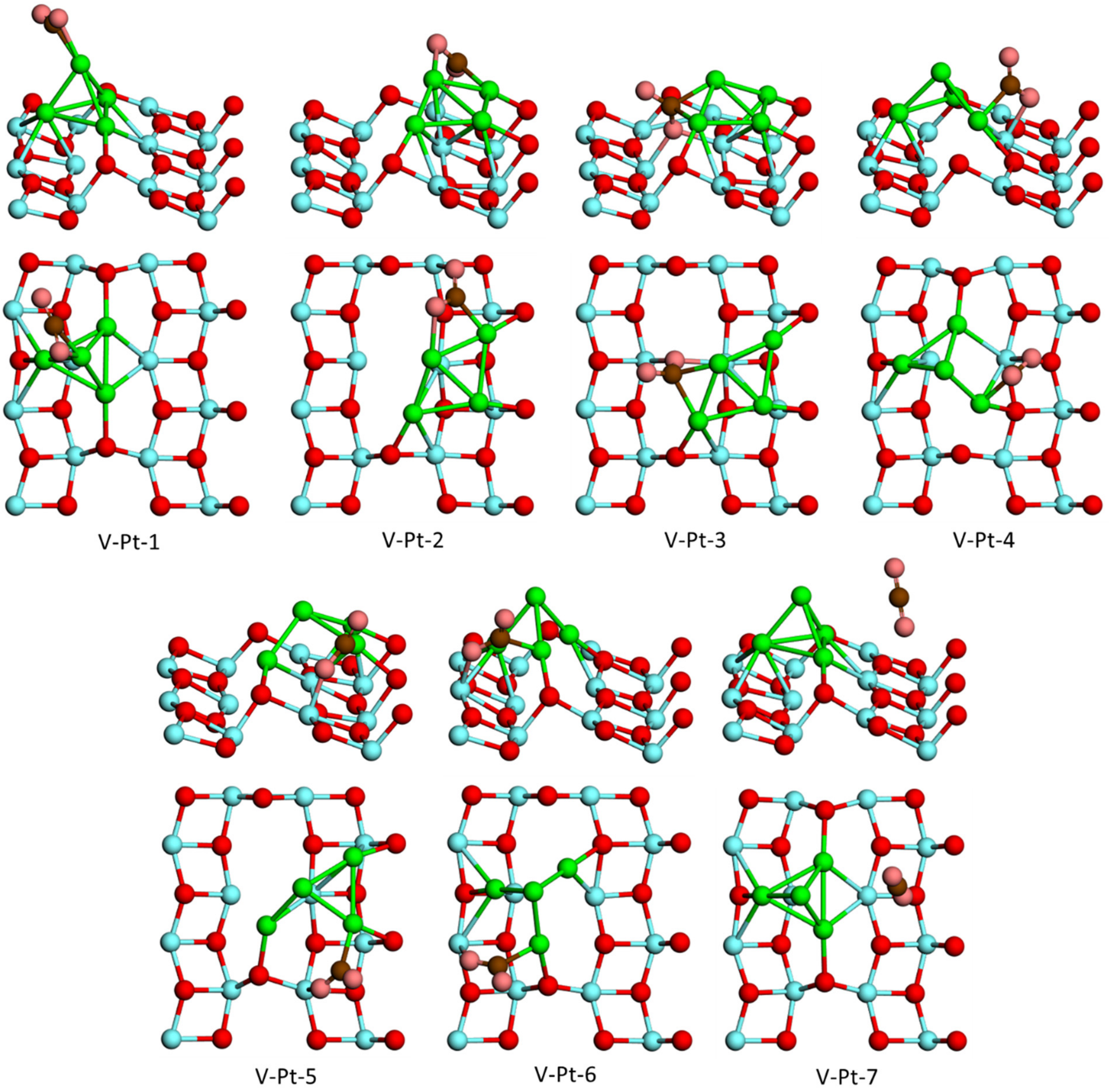

Figure S6 The geometry of CO_2 adsorption configurations on the V-Pt_4-TiO_2 structure. (Blue = Ti, Red = O, Brown = C, Pink = O of CO_2, and Green = Pt)

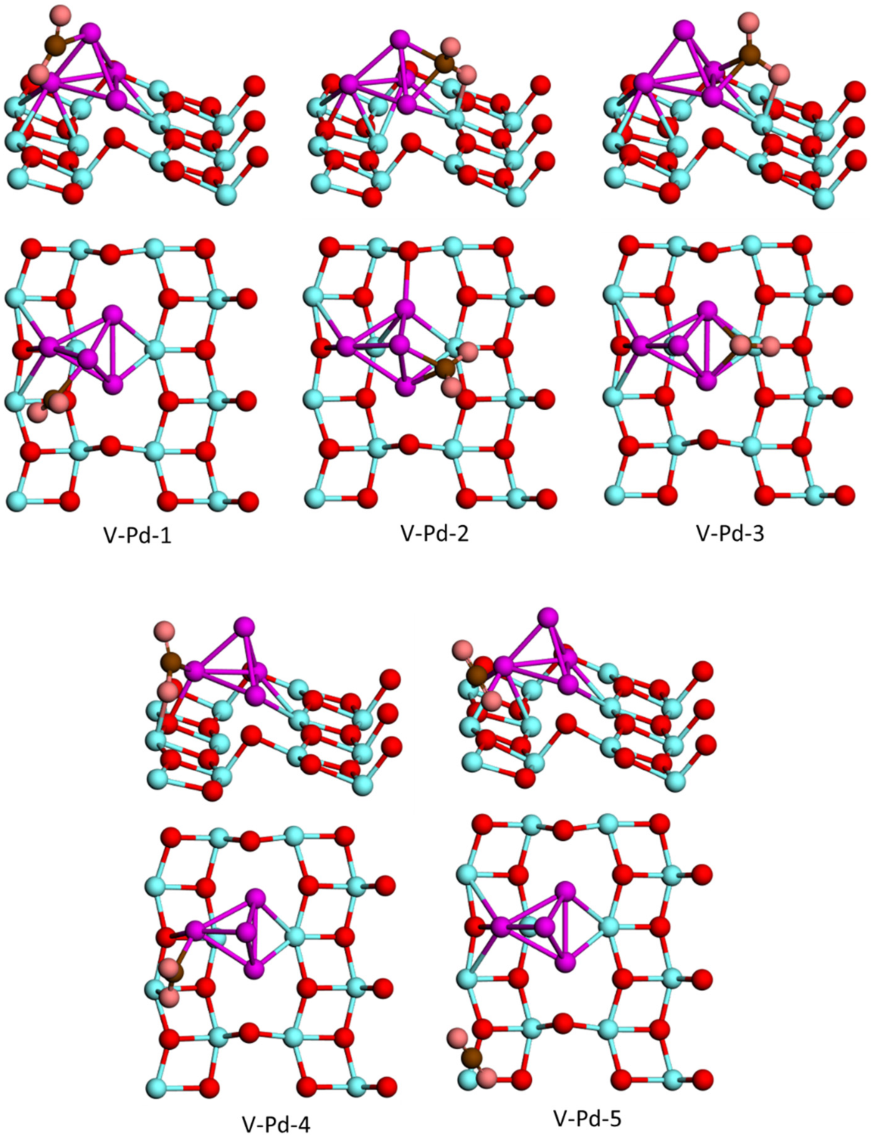

Figure S7 The geometry of CO_2 adsorption configurations on the V-Pd_4-TiO_2 structure. (Blue = Ti, Red = O, Brown = C, Pink = O of CO_2, and Purple = Pd)

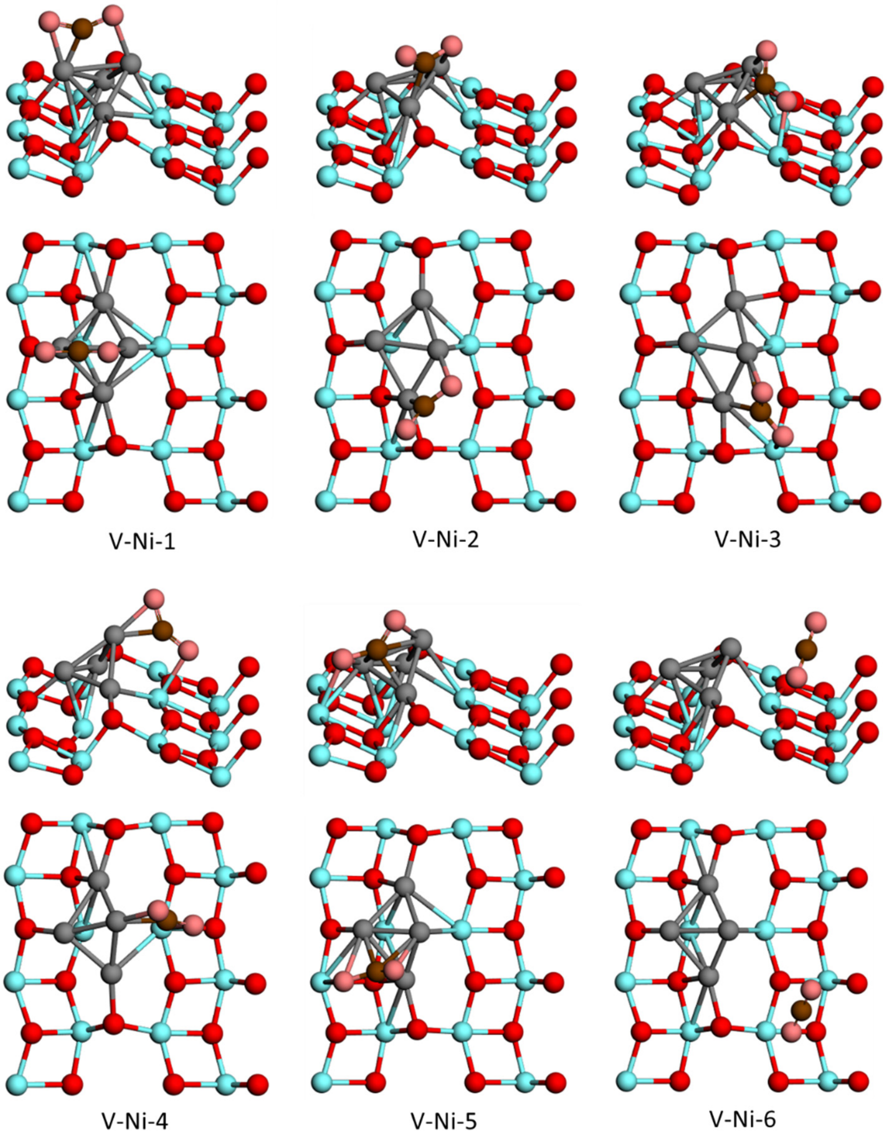

Figure S8 The geometry of CO_2 adsorption configurations on the V-Ni_4-TiO_2 structure. (Blue = Ti, Red = O, Brown = C, Pink = O of CO_2, and Gray = Ni)

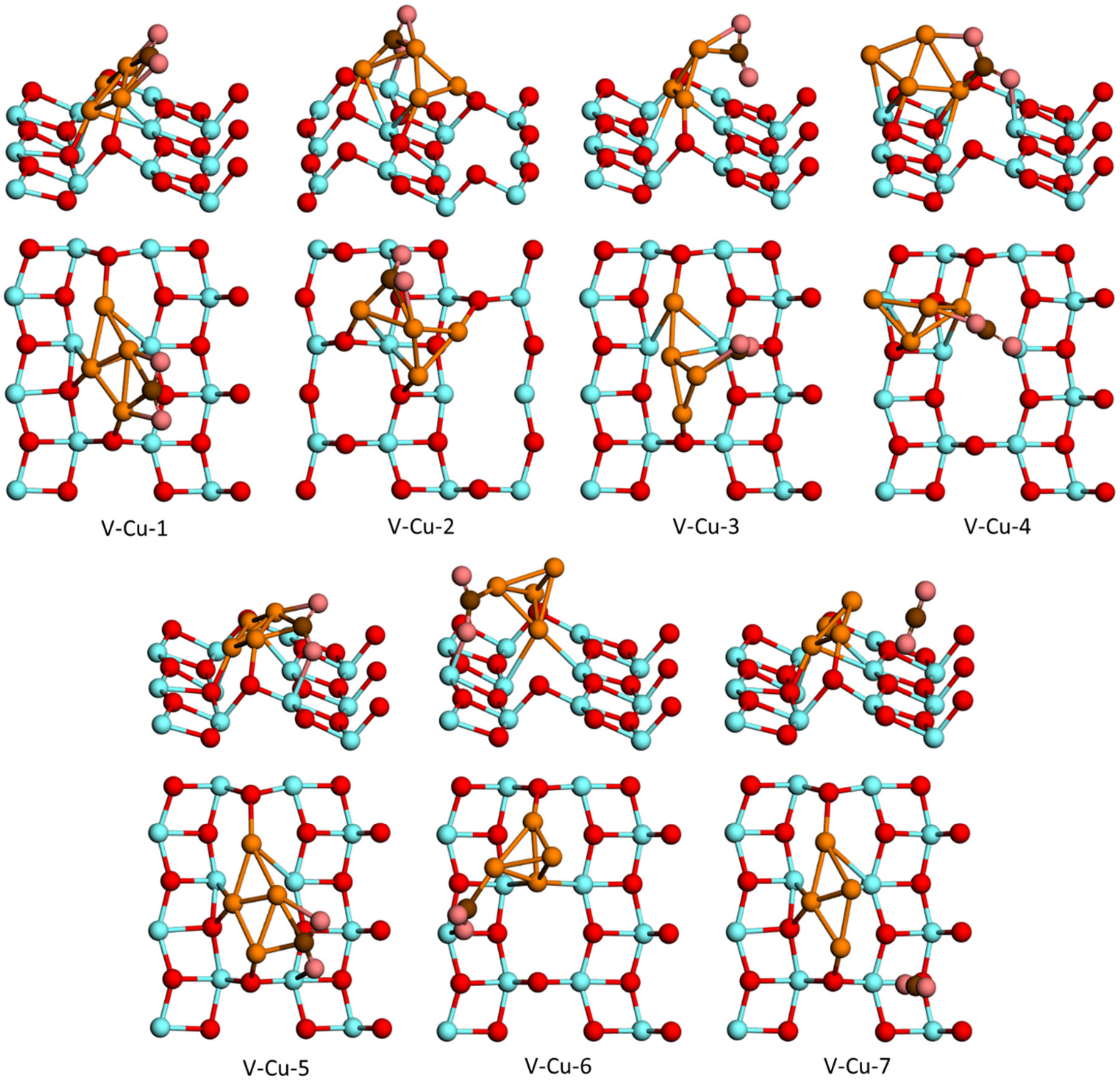

Figure S9 The geometry of CO_2 adsorption configurations on the V-Cu_4-TiO_2 structure. (Blue = Ti, Red = O, Brown = C, Pink = O of CO_2, and Orange = Cu)
